# Targeted splicing therapy: new strategies for colorectal cancer

**DOI:** 10.3389/fonc.2023.1222932

**Published:** 2023-08-17

**Authors:** Yifeng Zheng, Guoqiang Zhong, Chengcheng He, Mingsong Li

**Affiliations:** Department of Gastroenterology, The Third Affiliated Hospital of Guangzhou Medical University, Guangzhou, Guangdong, China

**Keywords:** alternative splicing, colorectal cancer, splicing isoform, tumor-associated splicing variants, targeted splicing therapy

## Abstract

RNA splicing is the process of forming mature mRNA, which is an essential phase necessary for gene expression and controls many aspects of cell proliferation, survival, and differentiation. Abnormal gene-splicing events are closely related to the development of tumors, and the generation of oncogenic isoform in splicing can promote tumor progression. As a main process of tumor-specific splicing variants, alternative splicing (AS) can promote tumor progression by increasing the production of oncogenic splicing isoforms and/or reducing the production of normal splicing isoforms. This is the focus of current research on the regulation of aberrant tumor splicing. So far, AS has been found to be associated with various aspects of tumor biology, including cell proliferation and invasion, resistance to apoptosis, and sensitivity to different chemotherapeutic drugs. This article will review the abnormal splicing events in colorectal cancer (CRC), especially the tumor-associated splicing variants arising from AS, aiming to offer an insight into CRC-targeted splicing therapy.

## 1 Introduction

In the past 20 years, colorectal cancer (CRC) has been one of the most life-threatening malignant tumors. According to global data released by the American Cancer Society in the Journal of Clinician’s Oncology in 2023, CRC has the third-highest incidence and second-highest mortality rates of all tumors (1). For the treatment options for this disease, it is acknowledged that molecular targeted therapies can provide effective treatment solutions, especially for patients with advanced metastases. In the targeted therapy of CRC, although most drug targets (e.g. *EGFR*, *VEGF*, etc.) play an important role in the differentiation and metabolism of normal cells, drug administration claims that these drug targets cannot avoid their toxic effects on healthy tissues (2). Therefore, how we can maintain the regulatory effect of this molecule on normal cells while targeting and inhibiting them is the key to have a breakthrough in the molecular targeting therapy of CRC. In recent years, as the functions and mechanisms of splicing-related molecules in CRC have become clearer, targeted therapy using splice variants as targets has been developed, which shows a higher tumor specificity and offers the potential for a safer and controlled CRC-targeted therapy (3). Although splice variant targeted therapy is a new type of targeted therapy, it has very limited targets for clinical application, failing to meet the drug needs of patients at different stages of CRC. Therefore, what comes first is to study the function and mechanism of splice variants in CRC to explore and screen excellent drug targets to promote targeted therapy for CRC.

### 1.1 RNA splicing process

The studies on pre-mRNA splicing were first reported in 1977 (4, 5). RNA splicing is the process in which DNA is transcribed to form an initial/pre-mRNA (pre-mRNA/hnRNA) and then is sheared by a spliceosome to form a mature mRNA. The spliceosome is responsible for pre-RNA splicing, which is a large molecular complex composed of five small nuclear ribonucleic acids (snRNAs) and various proteins. These five snRNAs are named U1, U2, U4, U5, and U6, each of which can be associated with specific proteins, forming five small nuclear ribonucleoprotein particles (snRNPs). These snRNPs sequentially bind to the precursor mRNA during the splicing of introns, leading to the formation of a lariat structure and bringing the upstream and downstream exons closer together. Specifically, U1 and U2 snRNAs pair with the boundary sequences at the 5’ and 3’ ends of the intron, followed by the addition of U4, U5, and U6 to form a complete spliceosome. What is noteworthy is that at this stage, the intron bends to form a lariat structure, and the upstream and downstream exons gradually approach each other. Finally, the spliceosome rearranges its structure, releasing U1, U4, and U5, while U2 and U6 form the catalytic center for the trans-esterification reaction. Splicing factors (SFs) are a group of proteins that cooperate with the spliceosome to catalyze this core cellular function. And studies have shown that mutations in SFs can disrupt the expression ratios of small nuclear RNAs and impair spliceosome assembly (6). This can result in premature pathogenic termination of mRNA translation.

**Figure 1:**
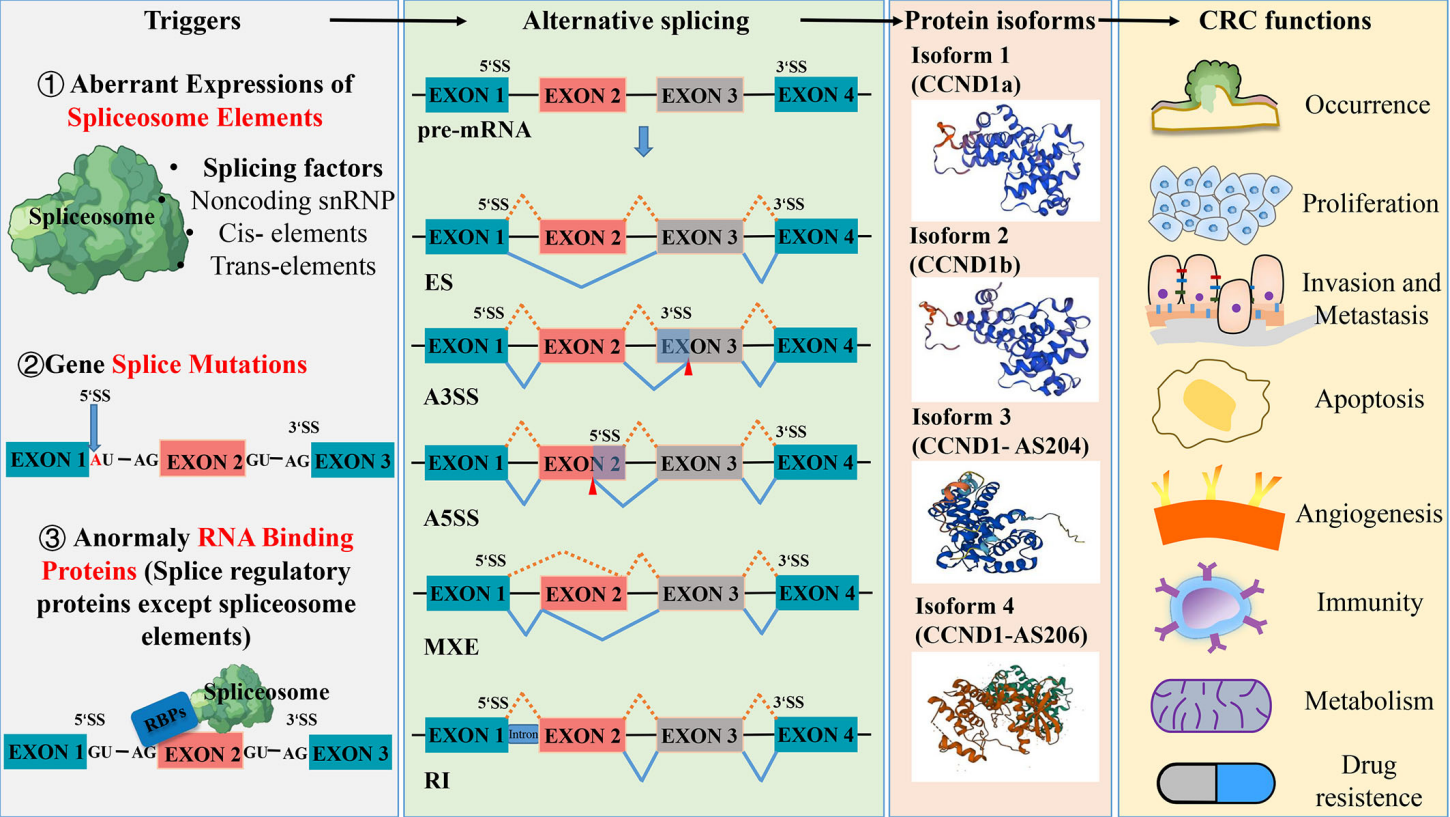
Aberrant splicing process in the occurrence and development of colorectal cancer. Abnormal spliceosome elements or gene splice mutation can trigger a variety of alternative splicing. The five common types of alternative splicing are exon skipping (ES), alternative 3’ splice site(A3SS), alternative 5’ splice site(A5SS), mutually exclusive exon (MXE) and retained intron (RI). These different types of alternative splicing result in the production of various protein isoforms, which can influence the function of colorectal cancer. Protein structures prediction using SWISS- MODEL (https://swissmodel.expasy.org/).

Alternative splicing (AS) has been regarded as one of the most important mechanisms that can maintain genomic and functional diversities since the Human Genome Project completed in 2004 (7). As a regulatory mechanism, AS affects almost all multi-exon genes in human body, in the sense that it allows multi-exon genes to produce more than one mRNA and generate multiple protein isoforms derived from the same single gene through differential sorting of exons. In this process, certain splicing patterns can cause loss or gain of key domains of proteins, leading to a lost or incomplete function, which in turn affects protein stability and changes subcellular localization. The type of AS includes intron retention, exon skipping, alternative 3’ splicing, and alternative 5’ splicing (**Figure 1**).

### 1.2 Alternative splicing and CRC

RNA splicing, which represents a crucial stage in gene expression, plays a pivotal role in regulating various aspects of cell proliferation, survival, and differentiation. Given this importance, abnormal changes in splicing events are closely related to the occurrence and development of tumors (3). The results of the deep mRNA sequencing of various tumor types have shown that cancer cells exhibit more complex and abnormal splicing behaviors compared to normal tissues (8), for instance, transcript ratios of cancer cells containing premature stop codons are significantly higher than the ones of normal tissues. Large-scale genome studies have discovered a series of splicing mechanisms that contribute to the development of tumors (9, 10), some of which can promote tumor growth by abnormal RNA splicing. For example, during the splicing process, abnormal changes in the copy number of splicing factors can produce more cancer-promoting splicing products (tumor-specific splicing variants) through alternative splicing (AS) and can promote the malignant growth and progression of tumor cells. Therefore, the abnormal expression of splicing factors is considered one of the direct causes of frequent and pathological splicing events in tumors (11). As the main process of tumor-specific splicing variants, AS can promote tumor progression by increasing the production of oncogenic splicing subtypes and decreasing the production of normal splicing subtypes, which is the focus of current research on the regulation of abnormal tumor splicing (12). Data from the analysis of 16 different tumors in the TCGA database show that almost all types of tumors exhibit abnormalities in intron retention, which is far more common than alterations in introns (13). In general, abnormal spliceosome elements or gene splice mutations can trigger various of AS, resulting in the production of different protein isoforms that have different functional effects on CRC (**Figure 1**). Capon et al. (14) were the first to discover that in CRC cell lines, c-Ki-ras (KRAS) mutates at different points within the same codon, resulting in the production of two transcript variants. So far, more than 15,000 alternative splices have been identified to be associated with various aspects of tumor biology, including cell proliferation and invasion, resistance to apoptosis, and sensitivity to different chemotherapeutic agents (15, 16).

### 1.3 CRC – targeted splicing therapy

Aberrant splicing is an important source that constitutes new cancer biomarkers, spliceosomes of which represent attractive drug targets for novel therapeutic agents. The research and treatment of tumor-specific splicing variants as new targets for CRC therapy have received extensive attention (7, 17, 18). Wang et al. (18) have discussed the association between various AS targets and the occurrence, progression, treatment, and prognosis of CRC. They argue that differential AS isoforms of the same gene may influence multiple biological functions in CRC, such as cell proliferation, metastasis, apoptosis, angiogenesis, immunity, and metabolism. Of the current targeted splicing therapeutic methods, oligonucleotide therapy is a relatively mature and widely used one in clinical practice, designed to alter splicing by Watson-Crick base pairing and hybridization to RNA in a sequence-specific manner. Clinical studies have shown that antisense oligonucleotides (ASO) can significantly reduce the mRNA that contributes to the survival of cancer cells. This therapy has achieved good results in correcting specific pathological splicing events in non-tumor single-gene diseases (19–21). Furthermore, small molecular compounds targeting splicing factors (e.g., RBM39) and splicing regulators have made progress in tumor treatment. Clinical studies have also reported strategies for combining splicing modulators with traditional antitumor agents to reduce their toxicity to healthy tissues (22, 23). In CRC-targeted splicing therapy, the current work focuses on exploring tumor-specific splicing variants which are expected to be diagnostic and prognostic markers of tumors. Some promising splice isoform targets have also been reported, including VEGF165b, c-FLIPL, CCND1b, etc. Thus, this article will review the tumor-associated splicing variants arising from AS, aiming to offer an insight into CRC-targeted splicing therapy.

## 2 Tumor-associated splicing variants in CRC: from roles to potential therapeutic approaches

**Figure 2:**
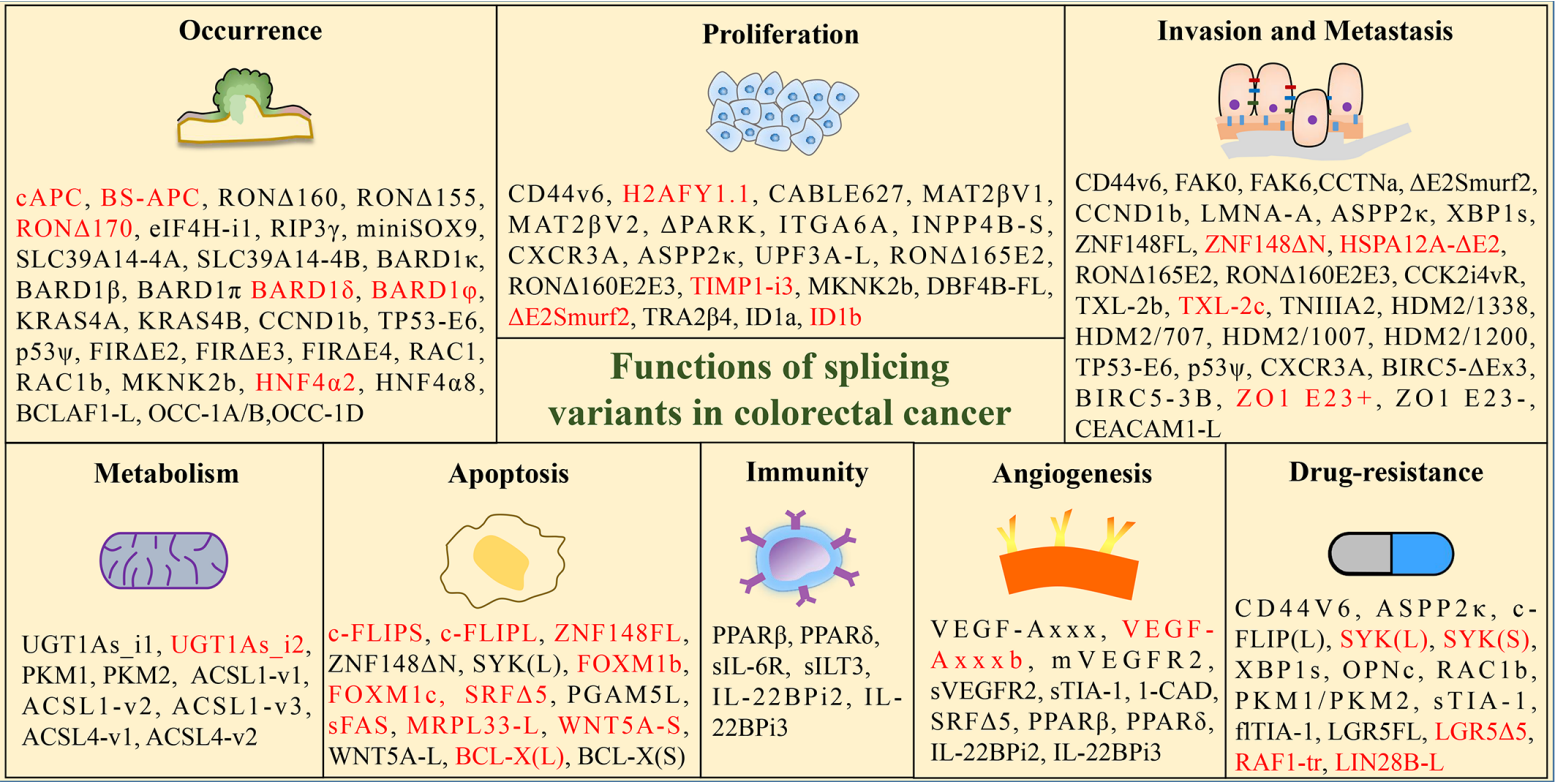
Functions of tumor-associated splicing variants in colorectal cancer. (Black font: promoting effect; red font: inhibition effect).

Investigating the influence of splicing variants in CRC is of paramount importance for the diagnosis and treatment of CRC. Subsequent paragraphs will elaborate on the function of splice isoforms in CRC by detailing its correlation with tumor initiation, progression, metastasis, immunity, metabolism, and drug resistance, shown in **Figure 2**.

### 2.1 Splice isoforms in the occurrence of CRC

The occurrence of cancer involves a complex process that has to do with the interaction of multiple genes and molecular pathways. Anomalies in alternative splicing have been identified as a significant contributor to the development of CRC, and studying this phenomenon has the potential to shed light on the mechanisms of tumor formation.

#### 2.1.1 RIP3

Receptor-interacting protein 3 (*RIP3*) is a member of the *RIP* family that induces apoptosis (24). Based on current research, RIP3 is known to be a crucial component of necrosomes and serves as an important mediator of inflammatory factors and infection-induced necroptosis (25). It has been implicated in promoting the occurrence and development of certain inflammatory cancer types, including pancreatic and colorectal cancers, by activating proliferation signaling pathways in cells and eliciting an immunosuppressive response within the tumor microenvironment (26).

Yang et al. reported two novel splice variants of human *RIP3*, named RIP3β and RIP3γ, which are generated by alternative splicing at the donor site of exon5 and retention of the intron between exons 5 and 6, respectively (27). Moreover, their study also revealed a significant increase in the ratio of RIP3γ to RIP3 in colon and lung cancer compared to their matched normal tissues, indicating that RIP3γ may be the primary isoform associated with tumorigenesis (27).

Existing evidence suggests that the widely used cancer treatments multi-targeting kinase inhibitors, such as Dabrafenib, Vemurafenib, Sorafenib, Pazopanib, and Ponatinib, also exhibit anti-necroptotic activity (28). This reveals the potential of targeting RIP3 in CRC for therapeutic interventions.

#### 2.1.2 APC

In colorectal tumors, the tumor suppressor gene *APC* (Adenomatous Polyposis Coli) is commonly found to be mutated (29). It produces various splicing isoforms associated with CRC tumorigenesis through abnormal splicing events such as exon skipping (e.g., exon 1, exons2-5, exon 7, exon 9A, exon 14, exon 10A) and intron retention (e.g., intron 11) (30). Three isoforms of the *APC* gene have been identified, namely cAPC, BS-APC, and 0.3 APC, resulting from alternative splicing of exon 1.

Previous studies have demonstrated that cAPC and BS-APC can effectively suppress the growth of colon tumor cells, while 0.3 APC lacks this effect. The loss of inhibitory function in 0.3 APC may be attributed to AS-induced changes in the conserved domain of the protein structure, which in turn impairs its ability to interact with other proteins (31). These results suggest that distinct *APC* isoforms may play different roles in the tumorigenesis of CRC.

#### 2.1.3 EIF4H

*EIF4H* (Eukaryotic Translation Initiation Factor 4H) encodes a translation initiation factor that stimulates protein synthesis by promoting mRNA utilization. Previous studies have indicated that *EIF4H* selectively regulates the translation of potent growth and survival factor mRNAs, thereby playing a vital role in translational control. This function can facilitate cellular transformation and has been implicated in cancer development (32).

The *EIF4H* gene is known to generate two splice variants, isoform 1 and isoform 2 through alternative splicing of exon 5 (33). Wu et al. discovered that the expression of EIF4H isoform 1 increased in CRC, and its overexpression in immortalized mouse fibroblast cells induced tumor formation in nude mice. Significantly, ectopic expression of EIF4H isoform 1 significantly increases the level of cyclin D1, while co-transfection of EIF4H isoform 1 siRNA and cyclin D1 expression vector can reverse the growth of the inhibitory effect of EIF4H isoform 1 knockdown (34). These findings suggest that EIF4H isoform 1 promotes the development of CRC through the activation of oncogenic signals and may serve as a potential therapeutic target for CRC treatment.

#### 2.1.4 BARD1

*BRCA1* Associated RING Domain 1 (*BARD1*), a binding partner of *BRCA1*, encodes a protein that interacts with the N-terminal region of *BRCA1* both *in vivo* and *in vitro* (35). Numerous studies have demonstrated that *BRCA1* plays a significant role in the onset and progression of colorectal cancer, with its mutations closely linked to CRC susceptibility (36–40). *BARD1* is necessary for the majority of *BRCA1’s* tumor suppressor functions, with *BRCA1*’s stability relying on its interaction with *BARD1* (41).

Through alternative splicing, *BARD1* can generate multiple isoforms, including BARD1κ, BARD1β, BARD1π, BARD1δ, BARD1φ, and others. Furthermore, the findings suggest that BARD1 isoforms κ, β, and π are associated with the occurrence and progression of CRC tumors and may serve as specific prognostic biomarkers. Conversely, isoforms δ and φ may have an inhibitory effect (42).

Recently, studies have found that poly ADP ribose polymerase (PARP) inhibitors selectively kill BRCA1-deficient cells by directly suppressing the fast recruitment of the *BARD1*-*BRCA1* heterodimer to DNA damage sites and impairing DNA repair. In addition, BARD1β has been demonstrated to enhance the sensitivity of CRC cells to poly PARP-1 inhibition, suggesting that it is a promising biomarker for assessing the suitability of homologous recombination targeting with PARPi in the treatment of advanced CRC (43).

#### 2.1.5 KRAS

The *RAS* (Rat Sarcoma Viral Oncogene Homolog) family is composed of small GTPases that are associated with the membrane and have critical functions in cell survival, proliferation, and differentiation (44). Central to cancer biology are the four proteins encoded by the three mammalian *RAS* genes, namely *HRAS* (Harvey Rat Sarcoma Viral Oncogene Homolog), *NRAS* (Neuroblastoma RAS Viral Oncogene Homolog), and *KRAS* (Kirsten Rat Sarcoma Viral Oncogene Homolog) (45).

In CRC, the *KRAS* gene is the most frequently mutated *RAS* gene (46). Alternative splicing of the *KRAS* transcript produces two variants with alternative 4th exons, which are referred to as KRAS4A and KRAS4B (47). When *KRAS* is constitutively activated by the mutation in exon 2 or 3, both KRAS4A and KRAS4B exhibit oncogenic properties (48). Furthermore, the direct regulation of hexokinase 1 by KRAS4A implies that the metabolic weaknesses of *KRAS*-mutant tumors may be influenced, at least in part, by the expression levels of the splice variants (49).

In a co-clinical trial conducted on RAS mutant colorectal cancer, the combined inhibition of MEK and CDK4/6 has been shown to exhibit therapeutic efficacy in patient-derived xenografts (50). Additionally, the trial has demonstrated the safety of Binimetinib and Palbociclib in patients with metastatic colorectal cancer with RAS mutations, identified biomarkers associated with treatment response, and revealed mechanisms of resistance that can be targeted (50).

#### 2.1.6 RON

The proto-oncogene receptor d’origine nantais (*RON, MST1R*) is a transmembrane tyrosine kinase receptor for macrophage-stimulating protein (MSP) that crucially regulates cell motility, adhesion, proliferation, apoptosis, and epithelial-to-mesenchymal transition (EMT) in various tumor biological processes.

The impact of *RON* on tumors arises from various splice variants generated by AS, including RONΔ170, Δ165, Δ160, Δ155, Δ110, and Δ55 (51). RONΔ160 is generated by skipping exons 5 and 6, while RONΔ155 is a derivative that lacks exons 5, 6 and 11 in combination, both of which can promote cell transformation and tumor growth (52, 53). In contrast, RONΔ170 can suppress the oncogenic activity of RONΔ160 in CRC cells, which is generated by skipping exon 19 (54). A constitutively active isoform generated by skipping exon 11, called DeltaRON, can activate epithelial-to-mesenchymal transition and increase the motility of expressing cells (55). Merestinib is an oral kinase inhibitor with antitumor proliferative and antiangiogenic activity developed initially to target the MET kinase. However, it has also shown the activity against other receptor tyrosine kinases, such as RON. While the safety and tolerability profile of Merestinib has been demonstrated, further investigation is necessary to determine its efficacy in targeting RON in CRC patients (56).

#### 2.1.7 CCND1

*Cyclin D1 (CCND1)* is a critical regulator of the cell cycle and is known to facilitate uncontrolled cellular proliferation, making it a key player in the development of cancer (57).

Research has shown that alterations in *CCND1* gene expression, including overexpression, underexpression, and variants, are associated with the development and poor prognosis of CRC (58–60), particularly the G870A mutation (60). This mutation is the most common splice mutation in *CCND1* (61, 62) and results in the generation of two *CCND1* isoforms through alternative splicing: full-length CCND1a and divergent C-terminal CCND1b (63, 64). It is widely accepted that an imbalanced CCND1a/b ratio or high expression of CCND1b is closely linked to the development of cancer. Recent studies have also revealed the role of CCND1b in cell cycle regulation, invasion, and metastasis (65, 66).

In terms of therapeutic strategies, research has shown that correcting *CCND1* splicing through antisense oligonucleotides (ASO) and small molecule modulators can be effective in cancer therapy (67). These findings suggest that developing splicing regulatory drugs targeting *CCND1* splicing variants could be a promising new option for the treatment of CRC.

#### 2.1.8 FIR

In colorectal cancer tissue, AS of the far-upstream element (FUSE)-binding protein (FBP)-interacting repressor (*FIR*) results in splicing variants that promote tumor development by disabling *FIR* repression, sustaining high levels of c-Myc, and opposing apoptosis (68).

Knockdown of SF3b, a subunit of SAP155 pre-mRNA-splicing factor, generates three splicing variants of *FIR*, including FIRΔexon2, Δ3, and Δ4. FIRΔexon2 lacks c-myc repression activity, and both FIRΔ3 and Δ4 are activated in human CRC tissue. This suggests that the overexpression of FIR and its splicing variants in CRC lead to the feed-forward or addicted circuit c-myc transcriptional activation (69). Furthermore, the combination of FIRΔexon2/FIR mRNA ratios with the real-time PCR detection of FIRΔexon2 mRNA significantly enhances the accuracy of screening for CRC, compared to conventional tumor markers CEA and CA19-9. Therefore, the mRNA expression of FIR, FIRΔexon2, FIRΔ3, and FIRΔ4 represents strong biomarkers for cancer screening (70). Spliceostatin A (SSA) exhibits anti-proliferative and anti-tumor activities by inhibiting spliceosome assembly through the nonproductive recruitment of U2 snRNP of subunit SF3b. Other compounds, such as meayamycin, pladienolide B, FD-895, and H3B-8800, can also interact with the SF3b subunit, thereby inhibiting the alternative splicing of *SAP155* (71, 72).

#### 2.1.9 RAC1

*RAC1* (Ras-Related C3 Botulinum Toxin Substrate 1), a small GTPase, is involved in various numerous dynamic cellular processes such as cell proliferation, cell survival, cell-cell interactions, EMT, cell mobility, and invasion (73–75).

The RAC1b variant is caused by the inclusion of exon 3b, resulting in the addition of a 19-amino acid sequence that is in-frame and located directly after the switch II domain. In addition, the equilibrium between RAC1 and RAC1b expression is modulated by splicing factors such as SRSF1 (76), hnRNP A1 (77), and SRp20 (78), which can promote or inhibit the inclusion of exon 3b *via* EGFR or Wnt signaling pathway (79). Experimental evidence indicates that RAC1b boosts G1/S progression and cell survival in NIH3T3 cells. Moreover, RAC1b may contribute to advanced stages of carcinogenesis, as it enhances Apc-dependent intestinal tumorigenesis and promotes carcinogenesis in the cecum and proximal colon during chronic inflammation (80).

Recently, highly effective and specific *RAC1* inhibitors have been discovered and developed, including GYS32661 and MBQ-167, which are currently undergoing preclinical trials for the treatment of advanced solid tumors (81). Therefore, due to its association with poor prognosis (82) and chemoresistance to oxaliplatin (83) of CRC, selectively targeting RAC1b and/or its interaction with molecular partners may represent a promising therapeutic approach for treating CRC.

#### 2.1.10 Others

Abdel-Samad et al. discovered that MiniSOX9, a truncated version of *SOX9* (SRY-Box Transcription Factor 9) lacking a transactivation domain due to the retention of its second intron, acts as an inhibitor of *SOX9*, suppressing the activity of the protein kinase Cα promoter and stimulating the classic Wnt pathway in CRC (84).

Thorsen et al. discovered that *SLC39A14*, a divalent cation transporter, undergoes the aberrant splicing in CRC tumor samples by mutually exclusive exon 4A and 4B, resulting in two splicing variants regulated by the Wnt pathway (85). Further studies found that the SLC39A14-exon4B transcript variant is a highly specific and sensitive cancer biomarker for colorectal tissue biopsies (86).

*TP53* (Transformation-Related Protein 53) mutations are frequently observed in CRC, and its splicing mutations can generate transcript variants with different tumorigenic and prognostic properties (87). Shirole et al. found that TP53 exon-6 truncating mutations produce the separation of the function of isoforms with pro-tumorigenic functions (88). Its function is similar to P53Ψ, a transcriptionally inactive P53 isoform, which can reprogram cells toward a metastatic-like state (89). In addition, an alternative P2 promoter located internally in intron 4 and the retention of intron 2, as well as alternative splicing of exon 9, can also lead to various splicing variants of TP53 and the loss of p53 activity (90). The various p53 proteoforms resulting from alternative splicing may aid in the early diagnosis of CRC.

Zhou et al. observed that the splicing factor SRSF10 is involved in the post-transcriptional splicing of Bcl-2-associated transcription factor 1 (*BCLAF1*) and forms the L isoform, thereby promoting the development of colorectal cancer (91).

*OCC-1* is considered as a differentially upregulated gene in CRC (92), which generates multiple splice variants through alternative splicing, including OCC-1A/B, OCC-1C, OCC-1D, and so on. The research findings indicate that the splice variants OCC-1A/B and OCC-1D of *OCC-1* can promote the occurrence of CRC by regulating the Wnt signaling pathway (93).

The nuclear receptor known as hepatocyte nuclear factor 4α (*HNF4α*) has been found to have tumor suppressive effects in the liver, but in colon cancer it appears to be amplified, suggesting an oncogenic role. *HNF4α* generates two splice variants, HNF4α2 (P1-HNF4α) and HNF4α8 (P2-HNF4α), through the use of two alternative promoters (P1 and P2) and two distinct 3’ splice events (94). The study indicates that HNF4α2 inhibits the development of colorectal cancer, while HNF4α8 has the opposite effect (95).

Before colorectal cancer develops into an advanced stage, it typically remains asymptomatic. Thus, it becomes crucial to identify additional risk factors in order to determine which segment of the population should undergo further colonoscopy. Various abnormal splice variants of genes have been proven to affect the occurrence of CRC. Furthermore, some genes such as BARD1 and HNF4α have splice variants that have completely opposite effects on CRC. Therefore, it can be inferred that targeting specific splice variants may be more effective and promising in comparison to targeting disease-causing genes. Further research on the genes and splice isoforms discussed in our previous review may lead to more advancements in the prevention, early diagnosis, and treatment of CRC.

### 2.2 Splice isoforms in the proliferation of CRC

It is a frequent occurrence for tumor cells to exhibit abnormal splicing activity, resulting in an elevated frequency of splicing isoforms that sustain abnormal proliferation and apoptotic patterns. Alternative splicing plays a role in the processes of proliferation, differentiation, and apoptosis by regulating the alternative expression of numerous oncogenic or tumor suppressor genes, as well as splicing factors.

#### 2.2.1 H2AFY

*H2AFY (MacroH2A1)* gene is a histone *H2A* variant that plays important roles in metabolic functions, transcriptional gene regulation, and DNA damage response (96).

*H2AFY* encodes two alternatively spliced variants, H2AFY1.1 and H2AFY1.2 (also known as MacroH2A1.1 and MacroH2A1.2), *via* mutually exclusive exon splicing (97). Novikov et al. observed that the percentage of MacroH2A1.1 relative to total MacroH2A1 was significantly reduced in CRC samples compared to normal controls, and the level of MacroH2A1.1 was regulated by QKI.

Moreover, the inhibition of proliferation mediated by MacroH2A1.1 is attributed to the decrease in protein levels of poly(ADP-ribose) polymerase 1 (PARP-1) (98). Multiple lines of evidence suggest that U2AF1 (S34F) can modulate alternative splicing, leading to a reduction in the MacroH2A1.1 isoform (97, 99–101).

#### 2.2.2 MAT2β

Methionine adenosyl transferase (*MAT*) is the sole enzyme responsible for catalyzing the formation of S-adenosylmethionine, which is the primary biological methyl donor (102).

Human methionine adenosyl transferase 2β (*MAT2β*) encodes two splice variants, V1 and V2, which differentially regulates cell growth. Of these, V1 plays a key role in the regulation of apoptosis and its knockdown has been shown to induce apoptosis in colon cancer cell lines (103). These two variants are present in both the nucleus and cytoplasm of colon cancer cells, and the overexpression of them can increase the levels of cytoplasmic HuR (an mRNA binding protein), thereby affecting cancer cell proliferation (104).

#### 2.2.3 ITGA6

Integrins consist of a heterodimeric pairing of an α and a β subunit. Currently, there are 18 α subunits and 8 β subunits that have been recognized, and they can combine together to create a total of 24 unique integrins (105).

During the formation of *integrin α6 (ITGA6)* subunit pre-messenger RNA, alternative splicing occurs to produce two distinct splice variants, namely integrins α6A (ITGA6A) and integrins α6B (ITGA6B) (106). These variants have different cytoplasmic domains, which contribute to their unique functions in cellular processes. Studies have suggested that the integrins α6A splice variant of the integrin α6 subunit in CRC cells plays a pro-proliferative role and activates the Wnt/β-catenin pathway to exert its effects (107). This pathway is recognized as the primary regulator of proliferative activity in the intestinal epithelium, both in its normal state and in CRC (108).

The study reveals that in CRC cells, the proto-oncogene MYC can control the activation of the promoter and splicing of the ITGA6 integrin gene through ESRP2 (109). This regulation promotes the production of the pro-proliferative ITGA6A variant. The pharmacological inhibition of MYC activity using the MYC inhibitor (MYCi) 10058-F4 leads to a decrease in the levels of ITGA6 and ITGA6A in CRC cells. This highlights the potential of targeted therapy against ITGA6A (109).

#### 2.2.4 UPF3A

*UPF3A*, also known as up-frame shift 3A, plays a role in both the NMD pathway and GCR. Specifically, it acts as an inhibitor of the NMD pathway while simultaneously promoting GCR (110).

Human *UPF3A* pre-mRNA is regulated by alternative splicing, which produces two splice variants, UPF3A-L and UPF3A-S. The two variants depend on whether exon 4 is included or excluded. These splice variants can give rise to two protein isoforms, UPF3A and UPF3A-S, which have distinct functions (111). Wang et al. discovered that knockdown of UPF3A-L inhibited the proliferation of CRC cells and induced DNA damage response and cell death. Furthermore, their study also found that CHERP and SR140, both identified as U2 snRNP-associated proteins, can regulate the splicing of *UPF3A* pre-mRNA by binding to the enhancer elements in exon 4 of UPF3A and activating its inclusion, thereby affecting the proliferation of CRC cells (112). The target gene of UPF3A is SRSF3, which is positively correlated with the expression of UPF3A. Increasing SRSF3 could enhance the invasion and metastasis of CRC cells, resulting in a poor prognosis. Targeted inhibition of UPF3A could reduce the genetic compensation response and offer a new therapeutic approach for treating CRC (113).

#### 2.2.5 MKNK2

Many kinase networks, such as EGFR, MAPKs, and c-Src, are involved in CRC development. *MNKs*, downstream of MAPKs, are protein kinases that can increase oncogenic mRNA translation by phosphorylating eIF4E, contributing to CRC pathogenesis (114).

MKNK2a and MKNK2b are two splice isoforms derived from the pre-mRNA of *MKNK2* through alternative splicing (115). The TCGA database showed that the MKNK2a/MKNK2b ratio was decreased in CRC tissues when compared to non-tumorous colon tissues (116).

Moreover, studies have found that CRC specimens exhibit decreased levels of MKNK2a and increased levels of MKNK2b, which are associated with *KRAS* mutations and tumor size. Their further experiments also demonstrated that elevated nuclear SRSF1 promotes MKNK2 splicing into MKNK2b rather than MKNK2a, thereby enhancing the proliferation of CRC tumors (117). SRPK inhibitors such as SRPIN340 and the PP1α-specific inhibitor Tautomycetin can efficiently disrupt SRSF1 phosphorylation, nucleus translocation, and MKNK2 alternative splicing (117). Therefore, this provides an opportunity for therapeutic intervention in CRC, such as the use of SRPK inhibitors or PP1α allosteric activators for the treatment of malignant tumors.

#### 2.2.6 Others

*CABLES* is a cell cycle regulatory protein that inhibits cdk2 activity by enhancing cdk2 tyrosine 15 phosphorylation by *WEE1*, ultimately leading to the inhibition of cell growth. However, research has revealed the presence of a 627bp abnormal splicing variant of *CABLES* in colon cancer, which leads to an increased cell growth rate in human colon cancer HT-29 cells, indicating that its role functions as a dominant negative mutant (118).

*PARKIN*, a tumor suppressor gene, functions as an E3 ligase and targets multiple substrates in the ubiquitin-proteasome system, inducing the degradation of cyclin E protein during the cell cycle. Its activity is modulated by growth factors. However, recent findings by Ikeuchi et al. have revealed that alternative splicing of the *PARKIN* gene leads to defects in the proteolysis of cyclin E, promoting colon cell proliferation and contributing to the development of colorectal cancer (119).

The 4-phosphatase Inositol polyphosphate 4-phosphatase II (*INPP4B*) is a regulator of the PI3K signaling pathway. The study demonstrated that a small transcript variant, INPP4B-S, generated by inserting a small exon between exon 15 and 16 and skipping exons 20-24, has been shown to promote the proliferation of colorectal cancer (120).

Flodrops et al. discovered that in CRC, tissue metalloprotease inhibitor I (*TIMP1*) increases proliferation and metastasis and decreases apoptosis by specifically regulating the FAK-PI3K/AKT and MAPK pathways. However, the splicing variant TIMP1-i3(+) generated by the retention of intron 3 of *TIMP1* is involved in inhibiting the progression of colon cancer during the early transition from normal mucosa to colorectal adenoma, and is regulated by hnRNPA1 (121).

It is established that *SMURF2* promotes the migration and invasion of cancer cells, indicating its potential oncogenic role in CRC (122). However, its splice variant ΔE2SMURF2 has been shown to control mouse intestinal tumor growth by upregulating the degradation of wild-type SMURF2 *via* type II TGF-β receptor and reducing the proliferation and production of pro-inflammatory cytokines (123).

The gene *DBF4B* produces two splicing variants, DBF4B-FL and DBF4B-S, through the inclusion or skipping of exon 6. Chen et al. found that the upregulation of SRSF1 promotes the inclusion of exon 6 in *DBF4B*, leading to the increased expression of DBF4B-FL and promoting the occurrence and proliferation of CRC (124).

The splice variant of the human transformer 2β (*TRA2B*) gene that contains exon 2 (*TRA2β4*) was found to be preferentially expressed in the nuclei of human colon cancer cells. It is possible that TRA2β4 could sequester Sp1 from binding to the promoters of target genes, which may promote cell growth by disrupting the gene expression program related to senescence (125). Nucleolin (126) and hnRNPA1 (127) have been shown to regulate the splicing of *TRA2β*, which affects the levels of TRA2β4 and is associated with the abnormal growth of CRC cells.

The expression of the inhibitor of differentiation 1 (*ID1*) was found to be positively correlated with high tumor grade in CRC patience (128). The *ID1* gene can generate two distinct isoforms through alternative splicing, known as ID1a and ID1b. Research findings indicate that the overexpression of ID1a promotes cell proliferation, while ID1b has the opposite effect by inhibiting proliferation and maintaining an undifferentiated cancer stem cell-like phenotype, as well as inducing cell quiescence (129).

*CDC14B* is an important regulator of mitotic spindle assembly in eukaryotes, which can have an impact on cancer cell proliferation and mitotic spindle dynamics. Matrin3 is a splicing regulator that can suppress the inclusion of exons 13 and 14 in the *CDC14B* mRNA. Since exon 13 contains a premature termination codon (PTC), knockdown of matrin3 can increase the formation of a CDC14B-PTC variant that inhibits the proliferation of CRC cells and promotes apoptosis. Therefore, the Matrin3/CDC14B axis represents a promising target for CRC treatments (130).

Sustaining proliferation is one of the malignant characteristics of the tumor growth. This process can be further enhanced by aberrant splicing and the consequent generation of oncogenic splicing isoforms. The aforementioned splice variants have all been shown to directly or indirectly impact the proliferation of CRC. In particular, the splicing isoforms of certain genes, such as H2AFY, TIMPI, SMURF2, and ID1, have been identified to possess inhibitory proliferation properties, indicating that therapeutic approaches targeting these variants would be highly beneficial for disease control and treatment in CRC patients.

### 2.3 Splice isoforms in the metastasis/invasion of CRC

Overcoming invasion and metastasis are critical challenges in treating CRC. The activation of EMT during cancer metastasis and recurrence is abnormal and relies on the interactions between cancer cells and the microenvironment. Accurately identifying whether a tumor is invasive or metastatic is crucial for determining its behavior.

#### 2.3.1 CD44

*CD44*, a transmembrane glycoprotein, can be alternatively spliced into multiple isoforms *via* the alternative splicing of its pre-messenger RNA (131). In the human gut epithelium, the presence of three isoforms, namely CD44s, CD44v6, and CD44v4-10, is commonly observed (132). Studies have indicated that CD44v6 has a negative impact on the prognosis of CRC patients, as it promotes CRC colonization, invasion, and metastasis, and even increases CRC cell resistance to anti-cancer therapies (133).

The good news is that several strategies targeting CD44v6 have been developed to date. Some strategies aim to block the interaction between HA and CD44v6, such as using the soluble CD44 ectodomain, α-CD44-HABD mAb, or the small fragment of HA (sHA). Other strategies mainly target the exon v6-encoded region by developing an α-CD44v6 mAb or by synthesizing a CD44v6-specific peptide (134, 135). Ejima et al. (136) recently have developed a novel anti-CD44 mAb, C44Mab-9, which can be utilized for detecting CD44v6 in various applications, and further research needed to determine whether C44Mab-9 has antitumor activity *in vivo*.

#### 2.3.2 CCTN

*CCTN (Cortactin)*, encodes an actin-associated scaffolding protein, is overexpressed in CRC and regulates cell migration (137). The *CCTN* transcript that contains exon 11, known as CCTN isoform-a, is the most abundant among all *CCTN* transcripts. This isoform is the wild type and dominant one, containing the full functional repeats, and has the strongest abilities in binding and cross-linking filamentous actin (F-actin) and promoting cell migration (138). In contrast, CCTN isoform-b and isoform-c (which are much less abundant) lack the 6th repeat (exon 11), resulting in a reduced F-actin binding and polymerization ability and significantly decreased cell migration when compared to CCTN isoform-a (138).

Studies have shown that as a potential functional RNA-binding protein, high levels of PTBP1 lead to the inclusion of exon 11 in the *CCTN* gene, promoting the generation of CCTN isoform-a and thereby enhancing cell migration and invasion in CRC (139).

#### 2.3.3 FAK

*FAK* is a type of cytoplasmic tyrosine kinase that is activated by both growth factors and integrins. Through AS of FAK pre-mRNA, specific exons (13, 14, 16, and 31) can be included independently, which in turn code for specific domains (boxes 28, 6, 7, and Pro-Trp-Arg, or PWR) that characterize *FAK* (140). There are different forms of *FAK* resulting from AS of its pre-mRNA. FAK0 is the most common form and is expressed in various tissues. FAK28 includes exon 13 and displays an increased expression with age, but its function in regulating FAK remains unknown. FAK6 and FAK7 include exons 14 and 16, respectively, and peak in expression during the final stages of embryonic development (141, 142).

The study found that FAK0 and FAK6 expressions are associated with metastatic potential in aggressive CRC cell lines HT29 and HCT116, suggesting that they could be markers of aggressiveness. FAK28 has a more specific role in tumor-microenvironment interactions. Therefore, FAK6 or FAK28 splice variants or their protein isoforms may be potential therapeutic targets for CRC primary tumors and metastasis (142).

#### 2.3.4 TNC

*Tenascin-C (TNC)*, encodes a matricellular protein, is abundantly expressed in both inflammatory lesions and tumor tissues (143, 144). Additionally, *TNC* contains a hidden functional site that consists of the amino acid sequence YTITIRGV, which is activated upon proteolytic cleavage (145).

Peptide TNIIIA2, a 22-mer *TNC* peptide that contains the functional sequence, has been found to strongly and persistently activate β1-integrins (146). The active sequence of TNIIIA2 is located within the cancer-associated alternative splicing domain, fibronectin type III repeat A2 (FNIII-A2), of the TNC molecule (147). Therefore, it is speculated that TNIIIA2-containing TNC peptides/fragments may play a role in cancer pathogenesis by inducing β1-integrin activation. OS2966 is a humanized and de-immunized monoclonal antibody that targets β1 integrin and has been shown to have antiproliferative, anti-invasive, antivascularization, and proapoptotic functions (148). This could be beneficial in CRC cases with high *TNC* expression.

Recent studies have demonstrated that peptide TNIIIA2 directly promotes the *in vitro* invasiveness of colon cancer cells by increasing the secretion of matrix metalloproteinase (149). Moreover, *in vivo* experiments using a spontaneous metastasis model have revealed that peptide TNIIIA2 is implicated in the metastasis of colon cancer cells to the lung (150). ST2146 is a biotinylated anti-tenascin monoclonal antibody and is a promising treatment for CRC (151).

#### 2.3.5 BIRC5 (SURVIVIN)

*BIRC5 (SURVIVIN)* is a member in the inhibitors of apoptosis (*IAP*) family regulating cell cycles and controlling programmed cell death (152). The human *BIRC5* gene comprises four dominant exons and two hidden exons. In addition to the wild-type *SURVIVIN*, alternative splicing of *SURVIVIN* pre-mRNA generates four different mRNAs that encode four unique proteins, namely SURVIVIN-ΔEx3, SURVIVIN-2B, SURVIVIN-3B, and SURVIVIN-2α (152, 153). Each splice variant has the potential to modulate survivin function by interacting with survivin during mitosis (154).

Ge et al. discovered that mRNA expression rates and levels of *SURVIVIN* and its four splice variants were increased in CRC tissues. Moreover, the expression levels of SURVIVIN-ΔEx3 and SURVIVIN-3B were positively correlated with tumor aggressiveness (153).

Currently, several *SURVIVIN* inhibitors are undergoing clinical evaluation, and more specific and effective *SURVIVIN* inhibitors are being developed. For instance, YM155 is a small-molecule inhibitor that specifically targets and suppresses the activity of the survivin promoter. LY2181308 and SPC3042 (EZN-3042) are antisense oligonucleotides that limit survivin expression by binding to and degrading its mRNA (155). The use of survivin-2B80-88 in combination with IFA and IFNα has also been shown to result in clinical improvement and enhanced immunological responses for patients with CRC (156). However, targeted drugs against *SURVIVIN* splice variants still require further discovery and investigation (157).

#### 2.3.6 CXCR3

The expression of C-X-C motif chemokine ligands (*CXCL*) 9, 10, and 11, along with other factors associated with EMT, is elevated at the invasive edge of CRC tissues (158). They involved in leukocyte trafficking, immune response, and cellular proliferation by binding to a common receptor, known as C-X-C motif chemokine receptor 3 (CXCR3) (159–161). This receptor belongs to the G protein-coupled receptor family and is expressed in CRC tissues (162).

In humans, three splice variants of *CXCR3* (CXCR3A, CXCR3B, and CXCR3-alt) have been discovered, and these variants play distinct roles in different types of cancer cells (163, 164). For example, gastric and renal cancer cells’ invasiveness and metastasis are promoted by CXCR3A (165), while prostate cancer cells’ invasiveness and migration are inhibited by CXCR3B (166). Recent studies have indicated that the CXCL10-induced proliferation and invasiveness of the HCT116 CRC cell line may be mediated by CXCR3A, not CXCR3B (167).

#### 2.3.7 FOXM1

The Forkhead box m1(*FOXM1*) is known to function as a transcription factor essential for G (1)/S transition and controls proper execution of mitotic cell division (168). It is a key mediator of Wnt/β-catenin signaling and acts by binding to β-catenin and stabilizing β-catenin in cell nuclear and enhancing transcriptional activity (169).

AS of exons 6 and 9 leads to the formation of various *FOXM1* isoforms. FOXM1a contains only exon 9, FOXM1b neither exon 6 nor 9, FOXM1c only exon 6 and FOXM1d contains both. FOXM1 is the inactive isoforms, while FOXM1b and FOXM1c remain functional (170). Recent study has shown that *AKT1* works as an upstream kinase, regulating RBM17-mediated FOXM1 alternative splicing and promoting the properties of cancer stem cells in CRC (171).

Rather et al. investigated the expression of *FOXM1* in 98 CRC samples and normal tissues, and found that *FOXM1* was elevated in CRC and linked to reduced disease-free survival (172). Overexpression of *FOXM1* in tumor tissues is also significantly related to metastasis in CRC through the induction of EMT (173). Another study also showed that the expression of *FOXM1* has a significant difference between CRC and adjacent noncancerous tissue samples. Silencing of *FOXM1* inhibited the proliferation, invasion, and migration of CRC cells. Furthermore, knockdown of *FOXM1* can also reduce VEGF-A levels in CRC cell lines, indicating that *FOXM1* could be a selective target for the molecularly targeted treatments of CRC (174). Additionally, SPF45/SR140/CHERP complex regulates *FOXM1* alternative splicing as well (170).

#### 2.3.8 Others

A-type lamins, which are produced by alternative splicing of the *LMNA* gene located on chromosome 1q21.3 (175), have been shown to increase the risk of death from CRC. This is attributed to their ability to enhance invasiveness and potentially induce a more stem cell-like phenotype (176).

Pan et al. made a discovery that SRSF11 plays a pro-metastatic role in CRC by impeding the AS of *HSPA12A* (Heat Shock Protein Family A (Hsp70) Member 12A pre-RNA). Their results highlight the novel connection between SRSF11-regulated splicing and CRC metastasis *via HSPA12A*, indicating that the PAK5/SRSF11/HSPA12A axis could serve as a promising therapeutic target and prognostic biomarker for CRC (177).

Multiple splice variants of the cholecystokinin-2 (*CCK2*)/gastrin receptor are ectopically expressed in gastrointestinal (GI) cancers. Studies have shown that one of these variants, CCK2i4svR, may enhance tumor angiogenesis through agonist-independent mechanisms, thus potentially contributing to the growth and metastasis of GI cancers (178).

*TXL-2*, a member of the thioredoxin (*TXN*) and nucleoside diphosphate kinase family, is a novel gene that undergoes alternative splicing to produce three distinct isoforms: TXL-2a, TXL-2b, and TXL-2c. Studies have demonstrated that TXL-2b significantly promotes cell invasion and metastasis through its interaction with the RAN and PI3K signaling pathways in CRC cells. In contrast, TXL-2c inhibits these processes (179).

The *HDM2* oncogene is known to negatively regulate the *P53* gene. In colorectal cancer tissues and cells, four *HDM2* splicing variants have been identified: HDM2/1338, HDM2/707, HDM2/1007, and HDM2/1200. Experimental results indicate that the expression of HDM2 splicing variants is associated with advanced tumor stage and distant metastasis in wild-type P53 cases, as well as poor survival of patients (180).

*ZO1* is a widely recognized cytoplasmic scaffolding and tight junction protein (181), and the AS event of ZO1 exon 23 (ZO1 E23) plays a crucial role in the progression of CRC. Research has shown that the deletion of ZO1 E23 (ZO1 E23-) leads to a disruption in F-actin distribution, which promotes CRC cell migration and invasion (182). Conversely, the inclusion of exon 23 in *ZO1* (ZO1 E23+) has the opposite effect. SRSF6 (183), HnRNP L (184), RBM47 (185), and GLTSCR1 (182) have all been shown to regulate ZO1 E23 AS, thereby impacting the development of CRC. The β2-adrenergic receptor agonist, indacaterol, has been identified as an inhibitor of SRSF6, which suppresses the AS of *ZO1* and subsequently suppresses CRC tumorigenesis (183).

Carcinoembryonic antigen-related cell adhesion molecule 1 (*CEACAM1*) is a protein that is often overexpressed in CRC and has been found to be correlated with clinical stage (186, 187). *CEACAM1* has alternatively spliced isoforms that contain either three or four Ig-like extracellular domains, and a long (CEACAM1-L) or a short (CEACM1-S) cytoplasmic tail (188). Studies have shown that compared to CEACAM1-S, CEACAM1-L promotes the invasion and migration of CRC (189).

In addition to the aforementioned variants, two splicing variants of *RON*, RONΔ165E2 (190) and RONΔ160(E2E3) (191), have been identified in recent years, and both have been demonstrated to enhance the growth and metastasis of CRC.

NF-Y is a heterotrimeric transcription factor composed of the DNA-binding subunit, NF-YA, and the histone-fold domain, NF-YB/NF-YC dimer. There are two splice variants of NF-YA: NF-YAs and NF-YAl. The latter results from the inclusion of exon 3 within the transactivation domain. Study has shown that high levels of NF-YAl transcription can forecast the poor overall survival in CRC patients, and tumor cells exhibiting elevated NF-YAl expression possess greater single-cell migratory and invasive potential. Targeting the NF-YAl splice variant and increasing the NF-YAs/NF-YAl ratio may decrease the progression of metastatic CRC (192).

The dissemination of tumor cells is the most dangerous process in the development of tumors. For many years, invasion and metastasis have been challenging obstacles in the battle against cancer, causing distress for both doctors and patients. Here, we have summarized some relevant splice variants and found that different splicing isoforms of HSPA12A, TXL-2, and ZO1 can promote or inhibit the invasion and metastasis process. Therefore, the discovery of splice variants associated with invasion and metastasis in CRC mentioned above brings new hope for effective treatment and improved prognosis in CRC.

### 2.4 Splice isoforms and the apoptosis in CRC

Apoptosis is a cellular process that occurs in physiological and pathological conditions, defects in apoptosis can lead to malignant transformation, tumor metastasis and drug resistance. AS of genes can impact the CRC development by affecting the apoptosis network of CRC.

#### 2.4.1 c-FLIP

FLICE-inhibitory protein (*FLIP*) is an inhibitor that regulates apoptosis mediated by death receptors (193). The Human *FLIP* gene is approximately 48 kb in size and includes at least 14 exons, which can generate 11 different isoforms through alternative splicing (194). Cellular FLIP (*c-FLIP*) is predominantly expressed as two splice variants, including a long form (c-FLIPL) with two serial death effector domains (DEDs) in the amino-terminal followed by a caspase-like domain (CLD) in the carboxy-terminal, and a short form (c-FLIPS) with only two N-terminal DEDs (194). Both splice variants of *c-FLIP* can inhibit proapoptotic downstream molecules (195).

c-FLIPL has been found to be significantly higher in colorectal cancer compared with matched normal tissue, suggesting that c-FLIPL may contribute to *in vivo* tumor transformation (196). The apoptosis induced by silencing of one splice form may be counteracted partly by the other splice form. However, researchers also found that specific silencing of c-FLIPL can effectively inhibit HCT116 tumor growth and induce apoptosis as silencing both splice forms, and c-FLIPL overexpression can dramatically inhibit the growth-inhibitory effects of chemotherapy *in vivo* setting, suggesting that the c-FLIPL may be the more important regulator of CRC (195).

#### 2.4.2 ZNF148

Zinc fingers proteins (*ZNF*) are the largest family of DNA binding proteins and can act as transcriptional factors in eukaryotes, and selectively binds to specific DNA sequences in the promoter of target genes *via* characteristic zinc finger domain (197). *ZNF148* plays an significant role in cell growth, proliferation, differentiation, apoptosis and other biological activities (198).

*ZNF148* has two functionally distinct alternative splicing isoforms. ZNF148FL contains a complete 794 amino acids, while ZBP-148ΔN was generated by alternative promoter usage upstream of an alternative exon 4B, and the ZBP-148ΔN protein lacks the amino-terminal 129 amino acids (197, 198). Two splicing isoforms of ZNF148 mutually antagonize with each other. Overexpression of ZNF148FL can decrease ZNF148ΔN expression, and promote the proliferation, migration, and invasion of human CRC cells trough binding to the transcription factor p300 and modulating the Wnt signaling pathway. On the contrary, overexpression of ZNF148ΔN can reduce levels of ZNF148FL and inhibit the upregulation of Wnt signaling pathway by ZNF148FL, subsequently promote the apoptosis, and inhibits the proliferation, migration, and invasion of CRC cells (198).

#### 2.4.3 SYK

Spleen tyrosine kinase (*SYK*) is a 72 kDa non-receptor tyrosine kinase that contains two tandem Src homology 2 domains at the NH2 terminus and a kinase domain at the COOH terminus (199, 200). *SYK* has two alternatively spliced isoforms: the full-length (SYK(L)) is predominantly found in nuclear, while the short form (SYK(S)) lacks a 69-nucleotide exon and is only expressed in the cytoplasm (199, 200). It has been shown that hnRNP-K protein regulates the splicing pattern of *SYK* (199).

*SYK* implicated in the control of apoptosis, and in the regulation of cell cycle. Deficiency of SYK (L) leads to the accumulation of cells in the G2-M phase of cell cycle, and to the emergence of cells with a >4N DNA (199). Ni et al. found that SYK (L) was downregulated in 69% of tumor tissue samples compared to the adjacent non-cancerous tissue, the expression of SYK (S) remained stable, suggesting that SYK (L) but not SYK (S) is associated with tumor suppressing activities (200). Denis et al. further demonstrated that survival of CRC cell depends on SYK(L), since silencing of SYK(L) expression affected cell viability and induced apoptosis (199, 200). C-13 is an original non-enzymatic inhibitor of SYK, which shows promising potential for the treatment of CRC and other cancer diseases (199).

#### 2.4.4 PGAM5

*PGAM5* is a member of the phosphoglycerate mutase family and has two splicing variants, including a long form (PGAM5L) and a short form (PGAM5S). Alternative splicing results in a truncation at amino acid residue 239 of the PGAM5 protein, with the PGAM5S isoform contains 16 additional C-terminal hydrophobic amino acids, while the PGAM5L isoform containing 50 additional hydrophobic amino acids residue at the C terminus (201, 202).

Both isoforms of *PGAM5* function in the intrinsic necrosis induced by TNF-α as well as reactive oxygen species (ROS) and calcium ionophore (201, 202). Further experiment indicated that PGAM5L is indispensable for the execution of intrinsic apoptosis by controlling the Bax activation and Drp1 dephosphorylation and induces mitochondria fission, Bax-PGAM5L-Drp1 complex is a potential target for CRC treatment (201).

#### 2.4.5 WNT5A

The canonical Wnt/β-catenin pathway is widely recognized as being associated with the formation of CRC (203). *WNT5A* (Wnt Family Member 5A) is an extracellular glycoprotein that activates Wnt signaling pathways, which are important in both development and tissue homeostasis (204, 205). According to a recent study, the opposing roles of *WNT5A* in cancer can be attributed to the encoding of two different splice isoforms, WNT5A-long (L) and WNT5A-short (S) (206). The WNT5A-L mRNA isoform can promote cell apoptosis, thereby suppressing cell proliferation and acting as a tumor suppressor in CRC cells. Conversely, the WNT5A-S mRNA isoform can inhibit cell apoptosis, promoting cell proliferation and playing an oncogenic role in CRC cells (207).

#### 2.4.6 Others

It is known that *FAS* (Fas Cell Surface Death Receptor) mediates apoptosis of CRC cells (208, 209). The pre-mRNA of *FAS* undergoes alternative splicing that excludes exon 6, resulting in the production of soluble FAS (sFAS) protein. This protein lacks a transmembrane domain and functions to inhibit FAS-mediated apoptosis (210).

The *MRPL33* gene is responsible for encoding a protein found in the large subunit of the mitochondrial ribosome. The depletion of MRPL33’s long isoform (MRPL33-L) which contains exon 3, has been shown to impair proliferation and increase apoptosis in both cancer cell lines and xenograft models (211). Studies have found that MRPL33-L expression is elevated in human colorectal cancer tissues, and this has been correlated with the levels of hnRNPK (211).

*BCL2L1*, a crucial gene in regulating apoptosis, is functionally involved in various cancer-related processes, and its protein expression has been linked to 20q gain. This suggests that the expression of *BCL2L1*, which is dependent on 20q gain, may play a role in the progression of colorectal adenoma to carcinoma. *BCL2L1* encodes two splice variants, an anti-apoptotic BCL-X(L) and a pro-apoptotic BCL-X(S) (212). ABT-737, a BCL-2/BCL-X(L) anti-apoptotic protein inhibitor, has successfully completed a prospective multicenter single-arm phase II study (213). This demonstrates the potential of targeting BCL-X(L) in CRC (colorectal cancer) therapy.

LINC00963 is an oncogenic lncRNA that is upregulated in CRC tissues. Recently, two novel variants of this gene, LINC00963-v2 and LINC00963-v3, have been discovered to be downregulated in CRC tissues. LINC00963-v2 lacks exons 2, 3, and 4, while LINC00963-v3 lacks exons 3 and 4. Overexpression of LINC00963-v2/-v3 in CRC cells has been found to suppress their proliferation, viability, and migration, and increase apoptosis. These effects are mainly due to attenuating the PI3K/AKT and Wnt/β-catenin signaling pathways. Therefore, these lncRNAs could serve as potential targets for CRC therapy (214).

Cancer typically inhibits the cellular apoptosis mechanism in the body, resulting in uncontrolled tissue growth. Chemotherapy uses the association between cellular apoptosis and cancer to destabilize the tumor and cause its death. It can be observed that the above-mentioned genes and their splice variants have different effects on apoptosis in CRC. Inducing the production of more pro-apoptotic splice variants could have a certain effect on the control and treatment of CRC.

### 2.5 Splice isoforms and the angiogenesis in CRC

Tumor growth, dissemination and metastasis are dependent on angiogenesis. AS of angiogenesis‐related genes can lead to the formation of distinct functional subtypes, while an imbalance among isoforms can impact tumor progression. It would be beneficial for the development and outcome of CRC if the regulation of splice variant proportions through targeting relevant splice variants could inhibit angiogenesis.

#### 2.5.1 VEGF

The vascular endothelial growth factor (*VEGF*) family of proteins regulates blood flow, growth, and function in both normal and diseased states, and VEGF-A is the most significant isoform of *VEGF* responsible for regulating angiogenesis (215). Additionally, VEGF-A and its receptors have been found to be highly expressed in mCRC (215).

The *VEGF* gene resides on chromosome 6 and consists of 8 exons (216). The *VEGFxxx* family of *VEGF* is produced through differential splicing in exons 6 and 7 and the proximal splice site in exon 8, whereas the distal splice site selection 66 bp downstream of the proximal splice site in exon 8 results in VEGFxxxb (217). The conventional VEGFxxx has angiogenic properties, while the VEGFxxxb isoform family has antiangiogenic properties, with xxx indicating the number of amino acids in a particular isoform (217). 12 isoforms of VEGF-A have been identified (218). The increase in VEGF-Axxx isoforms and the decrease in VEGF-Axxxb levels lead to an imbalance among the isoforms (215).

Bevacizumab is the first anti-angiogenetic treatment approved for clinical use in CRC patients. However, it has been reported to have a low response rate but a high rate of resistance and adverse events (219). Administering recombinant VEGF-Axxxb isoforms may be a promising new therapeutic approach (220).

#### 2.5.2 TIA-1

T‐cell Intracellular Antigen‐1 (*TIA‐1*) is a binding protein recognizing the complex secondary structure of the 3′ UTR, assisting in alternative RNA splicing, export and translational regulation that contribute to cancer formation and progression (221). *TIA-1* itself also undergo alternatively spliced in exon 6a to form two isoforms, namely flTIA-1 and sTIA-1 (222). TIA‐1 can bind to VEGF‐A RNA and act as a splicing and translational regulator of VEGF‐A, influencing the angiogenic capability of CRC (223).

sTIA-1 had been found to be highly expressed in *KRAS* mutant colon cancers. It exerts its effects by preventing flTIA-1 from inhibiting splicing and/or translating the VEGF-A165a, a pro-angiogenic isoform of *VEGF*, to promote tumor growth and angiogenesis (222). However, flTIA-1 expression also inhibited the effect of anti-VEGF antibodies, added a layer of intricacy to the anti-angiogenic treatment.

#### 2.5.3 CALD1

Caldesmon (CaD) is an actin-binding protein encoded by the *CALD1* gene. There are at least two high-molecular-weight isoforms (h-CaD) and four low-molecular-weight isoforms (l-CaD) produced by alternative splicing (224, 225). The alternatively spliced variants of the l-CaD are further differentiated by inclusion (Hela l-CaD) or exclusion (WI-38 l-CaD) of exon 1 (225).

The expression of Hela l-CaD was restricted to the tumor vasculature and was not found in normal blood vessels of cancers derived from colon and other various organs and was preferentially expressed in the early stage of tumor neovascularization. This indicates that Hela l-CaD can be considered as a marker of angiogenic endothelial cells during the early stages of tumor neovascularization (225). Kim et al. found that l-CaD significantly increases in primary colon cancer and liver metastasis than in the corresponding normal tissues, while h-CaD did not differ among these groups, and colon cancer patients with high levels of l-CaD had a poor response to chemoradiotherapy (226). These data suggested that l-CaD can be used for diagnosis and prognosis, and maybe a potential target for CRC treatment.

#### 2.5.4 VEGFR2

Vascular endothelial growth factor receptor 2 (*VEGFR2*) is the primary receptor of *VEGF*. There are two distinct forms of *VEGFR2* that are expressed: the membrane-bound VEGFR2 (mVEGFR2) and the soluble VEGFR2 (sVEGFR2) (227, 228).

Retention of intron 13 would lead to an in-frame early termination TAA codon, resulting in a truncated transcript variant. The protein product of this variant would lack the transmembrane and intracellular tyrosine kinase domains of *VEGRF2* (227). Tumor vascularization and tumor growth can be inhibited by both decreasing mVEGFR2 and increasing sVEGFR2 since sVEGFR2 has anti-angiogenic and anti-lymphangiogenic properties, whereas mVEGFR2 has the opposite effect (229).

Therapeutic drugs targeting *VEGFR2*, such as anti-VEGFR2 antibodies, siRNAs, and small-molecule *VEGFR2* inhibitors, have shown success in a variety of preclinical animal studies and clinical trials. Morpholino is considered a novel therapy that targets *VEGFR2* (229).

### 2.6 Splice isoforms and the immunity in CRC

Immune mechanism in tumor is very complex and is associated with AS. AS of genes can participate in the process of tumor immunity by affecting cytokine signaling or the function and infiltration of immune cells, which can impact tumor proliferation and migration. The discovery of immune-related splice variants associated with CRC will assist us in understanding the immune mechanisms of CRC and guide targeted and immunotherapy for CRC.

#### 2.6.1 IL6R

Interleukin-6 receptor (*IL-6R*) plays an important role in inflammation, immune cell differentiation and cancer. IL-6 can signal in two different ways, one is classic signaling *via* the membrane-bound IL-6R, another is trans-signaling *via* soluble forms of the IL-6R (sIL-6R).

sIL-6R can be generated from different mechanism, proteolytic cleavage of membrane IL-6R by transmembrane metalloproteases, release of cytokine receptors from cells on extracellular vesicles, and the generation an alternatively spliced mRNA isoform in transcriptional mechanism without the region encoding the transmembrane domain (230, 231). IL-6 can bind to IL-6R or sIL6-R to form the IL-6/IL-6R complex which can interact with the IL-6 transducer expressed gp130, subsequently results in gp130 dimerization and phosphorylation and activates the receptor-associated kinases such as JAK1, JAK2, and Tyk2, which eventually promote the cell proliferation and tumor progression (230). Recent studies have found a correlation between increased serum levels of IL-6 and sIL-6R in patients with CRC and tumor size as well as poor prognosis in those with metastatic colorectal cancer (230, 232). For instance, the compound Evodiamine has shown potential in inhibiting intestinal inflammation and the development of CRC by suppressing IL-6 signaling (233). These findings suggest that blocking IL-6 trans-signaling could play a role in the treatment of CRC.

Therapeutic drugs targeting IL-6R are currently under development. For example, Tocilizumab, an anti-IL-6 receptor antibody, has completed phase III randomized controlled trials (234), while Olamkicept, a soluble gp130-Fc fusion protein that selectively inhibits trans-signaling of interleukin-6 (IL-6) by binding to soluble IL-6 receptor/IL-6 complex, has completed randomized clinical trials (235).

#### 2.6.2 PPAR

Peroxisome proliferator-activated receptors (*PPARs*) belong to the nuclear hormone receptor family including three AS isoforms, namely PPARα, PPARβ/δ and PPARγ. PPARβ/δ-linked tumorigenesis was first identified in CRC and was considered as a potential drug target for CRC (236). The organization of the coding exons of PPARβ/δ corresponds to that of the genes encoding PPARα and PPARγ. PPARγ1 and γ2 are generated by using the differential promoter and AS (237), and four different splicing isoforms of PPARβ/δ mRNAs containing one or two non-coding 5’-exons are also generated by alternative promoter (238).

*PPAR* can promote lipid accumulation in NK cells, inhibit of their cellular metabolism and thus inhibit their function (239). Schumann et al. found that most of PPARβ/δ target genes are upregulated in tumor-associated macrophages (TAMs) from ovarian carcinoma patients, activation of PPARβ/δ target genes by polyunsaturated fatty acids which act as potent PPARβ/δ agonists in macrophages contributes to the pro-tumorigenic polarization of ovarian carcinoma TAMs (240). Therefore, PPARβ/δ has the pro-tumorigenic functions by promoting polarization of macrophages favoring tumor progression or impairing antitumor cytotoxicity of NK cells (241). A recent study has found that blocking the PPAR pathway can promote apoptosis and inhibit the development of CRC organoids *in vitro*, indicating that the PPAR signaling pathway is involved in CRC tumorigenesis (242).

#### 2.6.3 IL22RA2

Interleukin-22 (IL-22) is an IL-10-type cytokine involved in various pathologic processes. It is signaled through a membrane receptor composed by the heterodimer IL-22R1/IL-10R2 and can be recognized by a secreted receptor called IL-22 binding protein (IL-22BP), which is encoded by the *IL22RA2* gene (243). Human *IL22RA2* gene can express three alternatively spliced variants including IL22RA2v1 (IL-22BPi1), IL22RA2v2 (IL-22BPi2), and IL22RA2v3 (IL-22BPi3), IL-22BPi1 was retained intracellularly because of the presence of exon 3 in its mRNA; the sequences of IL22RA2v1 and IL22RA2v2 differ only in exon 3; IL-22BPi2 consists of two fibronectin III domains, whereas IL-22BPi3 lacks the C-terminal domain except for five frameshifted residues (244).

IL-22BP is highly expressed by dendritic cells (DC) in colon under homeostatic conditions and plays a crucial role in controlling tumorigenesis and epithelial cell proliferation. Although IL-22BPi3 was more abundant in human tissues, IL-22BPi2 was more effective than IL-22BPi3 at blocking IL-22 signaling, while IL-22BPi1 was unable to antagonize IL-22 signaling because it is not secreted (244).

IL-22BP deficiency can lead to the accelerated and increased tumorigenesis in colitis-associated colon cancer model (245). However, it is also reported that CD4+ T cells from patient with IBD produce high levels of IL-22BP, which can block the protective actions of IL-22 during acute colitis (246, 247). A study demonstrated that the delivery of liposome-protamine-IL-22BP mRNA complex can induce tumor apoptosis, inhibit angiogenesis, and increase infiltration of immune cells, showing a promising potential for colon cancer therapy (248).

#### 2.6.4 ILT3

Inhibitory receptor Ig-like transcript 3 (*ILT3*) is an immunoregulatory protein that belongs to the *ILT* family. Human *ILT3* is mainly expressed in dendritic cells and monocytes. It is generally viewed as having a negative regulatory function (249).

Alternatively spliced mRNA that results from the deletion of exons 5–7 of *ILT3* encodes a soluble form of the ILT3 (sILT3) protein, which lacks the ILT3 transmembrane domain, causing the release of ILT3 in the circulation (250). Both membrane-bound ILT3 and sILT3 could inhibit the proliferation of T cells, induce its anergy, and promote the differentiation of CD8+ T cells within the tumor microenvironment or in sentinel lymph nodes. Furthermore, patients with CRC have been found to have a significantly higher amount of sILT3, which inhibit tumor immunity in CRC (250). A study revealed that the decreased expression of ILT3 in CRC patients is associated with improved overall survivals (251). The data suggested that the expression of *ILT3* could have a significant impact on the progression of CRC and serve as a target for individualized therapy.

### 2.7 Splice isoforms and the metabolism reprogramming in CRC

Metabolic reprogramming is a distinguished cancer hallmark. AS can affect CRC through participating in many metabolic pathways, such as lipid metabolism and carbohydrate metabolism. Studying the genes and their splice variants associated with CRC metabolic reprogramming can aid in the development of new treatment strategies, such as targeting these variants to interfere with the survival and proliferation of cancer cells by disrupting their metabolic pathways. This has the potential to become an important approach in future cancer therapy.

#### 2.7.1 PKM

The Warburg effect is characterized by the preference of tumor cells for glycolysis over oxidative phosphorylation for energy production, and this metabolic shift is a crucial factor in malignant transformation. Studies have shown that this metabolic alteration results from a change in the expression of different splice variants (PKM1 and PKM2) of the glycolytic enzyme pyruvate kinase (PK) (252). The PKM1 isoform promotes oxidative metabolism, whereas PKM2 enhances aerobic glycolysis. And data suggest that the decrease in PKM1 expression may contribute to the upregulation of glycolysis and the downregulation of butyrate oxidation in CRC cells (253). Furthermore, multiple studies have suggested that PTBP1 (254), lncRNA SNHG6 (255), lncRNA HOXB-AS3 (256), Sam68 (257), MicroRNA-124 (258), LncRNA XIST/miR-137 axis (259), TRIM29 (260), and other molecules can target PKM1/PKM2 and influence their ratio, thereby impacting the growth, glycolysis, and even chemoresistance of CRC cells.

#### 2.7.2 UGT1A

UDP-glucuronosyltransferase enzymes (UGTs) are responsible for glucuronidation pathway which is a major cellular process of conjugative metabolism (261). Girard et al. (262) found that a new exon 5b, located in between the coding exons 4 and 5, can undergo alternatively spliced with exon 5a (the classical exon 5), generating new UGT1A mRNA variants referred to as isoforms 2 or i2. UGT1A_i2 is enzymatically inactive and acts as a negative modulator of UGT1A1_i1, resulting a significant repression of UGT1A_i1-mediated drug metabolism (262, 263), and influencing cancer cell metabolism *via* complex protein network connecting other metabolic pathways (264). Studies have shown that UGT1A_i2 mRNA is downregulated in colon tumors, and the depletion of UGT1A_i2 proteins in colon tumors cell model can enforce the Warburg effect, leading to lactate accumulation and impacting migration properties (264).

#### 2.7.3 ACSL

Long-chain acyl-CoA synthetases (*ACSL*) plays a crucial role in the degradation of fatty acids, the remodeling of phospholipids, and the synthesis of long acyl-CoA esters that controls a multitude of physiological processes in mammals. Five *ACSL* genes have been identified, namely ACSL1, ACSL3, ACSL 4, ACSL 5, and ACSL 6, with each gene having up to five different spliced variants, and most spliced variants are generated by AFE, ES, and MXE (265). Among these spliced variants, ACSL1 and ACSL4 were found to be overexpressed in CRC patients with poorer outcomes (266).

The metabolic profiles of both ACSL1 and ACSL4 isoforms were significantly different. ACSL1 was more inclined to triglyceride synthesis while ACSL4 prefers longer polyunsaturated fatty acids (PUFA) such as arachidonic acid as substrates. Furthermore, ACSL1 exhibits a tendency towards invasive capabilities accompanied by a decrease in the basal oxygen consumption rate, whereas ACSL4 promotes the proliferation in CRC cells and is related to a more glycolytic phenotype compared to control or ACSL1 cells (266). It is reported that the combination of ACSL/SCD inhibitors can reduces the survival of CRC cells without impacting normal cells, and it is also effective in CRC cells resistant to the conventional chemotherapy. Therefore, the inhibition of ACSL/SCD axis is of great potential in cancer treatment (267).

### 2.8 Splice isoforms and the drug resistance in CRC

AS can not only influence therapeutic efficacy but also serve as a prognostic and predictive biomarker for CRC. Different AS isoforms may have contrasting functions in drug resistance. Targeting these isoforms is highly likely to help adjust and refine the corresponding treatment strategies, overcome cancer drug resistance, and thus improve the therapeutic efficacy of CRC.

#### 2.8.1 ASPP2

*ASPP2* is a tumor suppressor that enhances apoptosis and inhibit tumorigenesis *via P53*-dependent and *P53*-independent pathways (268, 269). Exon-skipping splicing of *ASPP2* results in the generation of ASPP2κ, which is a C-terminally truncated isoform that lacks the *P53* binding sites. This isoform is defective in promoting stress-induced apoptosis (270). The overexpression of ASPP2κ in tumor tissue compared to adjacent normal tissue contributes to CRC by enhancing proliferation, promoting cell migration, and conferring resistance to chemotherapy-induced apoptosis (271). It serves as a potential treatment target and acts as a prognostic and predictive biomarker for CRC.

#### 2.8.2 OPN

Osteopontin (*OPN*) is an extracellular matrix protein that is overexpressed in various cancers. It promotes cancer cell proliferation, survival, metastasis, and angiogenesis. There are three main splicing isoforms of *OPN*: OPNa, OPNb, and OPNc. OPNa is the full-length wild-type form, while OPNb and OPNc are mutually exclusive splicing isoforms. OPNb lacks exon 5, while OPNc lacks exon 4 (272). After 5-FU treatment of colon cancer cells, the splicing isoform OPNc was found to be the most upregulated in comparison to the other two isoforms, and the secretory OPNc can stimulate cells to survive from drug-induced microenvironmental stress (273). Preventing *OPN* splicing could be an effective method of inhibiting tumor progression and recurrence.

#### 2.8.3 LGR5

*LGR5* can inhibit the degradation of β-catenin, resulting in the accumulation of β-catenin and its translocation into the nucleus where it regulates the expression of a wide range of target genes (274). *LGR5* has been reported to be overexpressed in CRC patients and correlated with poor prognosis (275). Additionally, *LGR5* has been found to drive tumorigenesis in both the small intestine and colon (276).

*LGR5* consists of 18 exons, with exons 1–17 constituting extracellular leucine-rich repeats (LRRs). There are two transcript variants of *LGR5*, one lacking exon 5 (LGR5Δ5) and the other lacking exon 8 (LGR5Δ8) (277).

LGR5FL-positive cells exhibit low proliferative activity and resistance to anti-tumor drug, while blocking *LGR5* exon 5 impairs the dormancy of LGR5FL-positive cells and gives the ability of proliferation, subsequently increasing the sensitivity to chemical treatments (277). The study has also demonstrated that the low level of LGR5Δ5 expression was significantly correlated with a poor prognosis for the disease-associated survival of soft-tissue sarcoma patients (278). It appears that the *LGR5* exon 5 Ab has the potential to be a new and promising drug for CRC.

#### 2.8.4 Others

*SYK* is associated with the survival of CRC cells. Although the overexpression of SYK(S) did not alter proliferation and metastasis, SYK(S) is important in the chemotherapeutic treatment of CRC, Both SYK(L) and SYK(S) can increase the sensitivity of CRC cells to 5-FU, which is significant in cancer treatment (200).

AS of *FOXM1* leads to its functional isoform and promotes 5-FU resistance by upregulating ABCC10 through directly binding to its promoter region, silencing of *FOXM1* promoted the sensitivity of CRC cells to 5-FU by enhancing cell apoptosis (170, 279). The study has also demonstrated that *FOXM1* can potentially regulate other 5-FU targets, such as thymidylate synthase (TYMS), thymidine kinase 1 (TK-1) and thymidine phosphorylase (TYMP); inhibiting *FOXM1* leads cell cycle arrest, DNA damage, and apoptosis in CRC cell lines (280).

Alternative splicing results in the inclusion of a new exon 11 in the *RAF1* mRNA, which causes a frameshift and introduces three premature stop codons, leading to the truncation of the RAF1 protein and the absence of its C-terminal kinase domain, The resulting splice isoform is named RAF1-tr (281). RAF1-tr can increase nuclear localization and inhibits the function of DNA damage–regulating protein. This leads to an increase in the levels of DNA damage after the exposure to bleomycin and radiation, and enhances the apoptotic response of CRC cells to double-stranded DNA damage (281).

The unfolded protein response (*UPR*) is a cellular stress response related to the endoplasmic reticulum (ER). Inositol requiring enzyme 1 (IRE1α) is a ER-localized proteins that constitutes one arm of the *UPR* (282). Chemotherapeutic agents trigger ER stress and activate *UPR*. Upon activation, IRE1α removes a 26-bp nucleotide intron from the mRNA encoding X-box binding protein (XBP) 1 to causing a frame-shift and producing an active form XBP1s, which controls the expression of genes involved in protein folding, ER-associated degradation, protein quality control and phospholipid synthesis (282–284). Sustained activation of the *UPR* contribute to oncogenic processes, metastasis, and tumor chemotherapy resistance (282, 285).

*LIN28B* has two alternative splicing isoforms which are different in 5’ exons, namely the LIN28B-long and LIN28B-short isoforms. The LIN28B-long isoform consists of 250 amino acids and has both cold shock domain (CSD) and zinc finger domains (ZFDs), whereas the LIN28B-short isoform lacks 70 amino acids in the N-terminus and deficient with a complete CSD (286, 287). The overexpression of LIN28B-long isoform can downregulate LET-7 expression, which negatively regulates the RAS/ERK signaling. The LIN28B-short isoform does not suppress LET-7 and acts as an antagonist against the LIN28B-long isoform in normal colonic epithelial homeostasis (287). Therefore, it is the LIN28B-long isoform rather than the LIN28B-short isoform that contributes to the drug resistance. Targeting the CSD of *LIN28B* may have a potential therapeutic effect in treating *LIN28B* positive CRC.

CACClnc is a recently discovered novel lncRNA. It can promote drug resistance in CRC by specifically binding to YB1 and U2AF65, both of which are splicing factors. This binding promotes their interaction and then modulates the AS of RAD51 mRNA, thereby promoting DNA repair and enhancing homologous recombination (288). Targeting CACClnc and its associated pathway may assist in improving treatment outcomes for CRC patients with chemoresistance.

## 3 Conclusion and perspectives

In summary, under the influence of various factors, abnormal splicing events in genes lead to the generation of different splicing variants. Due to distinct coding information, these isoforms can encode proteins with different structural and functional characteristics, in the sense that they may impact the activity of signaling pathways, regulate the cell cycle, and affect the stability of genes, thereby exerting different functional effects on CRC. For instance, when compared to ZNF148FL, ZNF148ΔN is generated through alternative promoter usage upstream of an alternative exon 4B. Consequently, it lacks the amino-terminal 129 amino acids, part of the transcriptional activation domain of the protein. This difference leads to the mutually antagonistic effects and distinct roles in the development of CRC. Different from typical targeted therapies, targeted splicing therapy usually has higher tumor specificity due to acting on abnormal splicing events in tumors. Thus, targeted splicing therapy is expected to achieve the targeted inhibition of cancer-promoting molecules while maintaining the regulatory effect of the molecule on normal cells and reducing the impact on healthy tissues for traditional antitumor drugs cannot avoid side effects and toxicity. In other words, targeted therapies are supposed to substitute these traditional drugs. As a more effective and safer new strategy for tumor treatment, targeted splicing therapy has great potential for the development in the field of oncology treatment (e.g. CRC). Because different splicing events occur in different phenotypes of CRC, personalized targeted splicing treatments are necessary to improve outcomes and minimize adverse effects.

**Figure 3:**
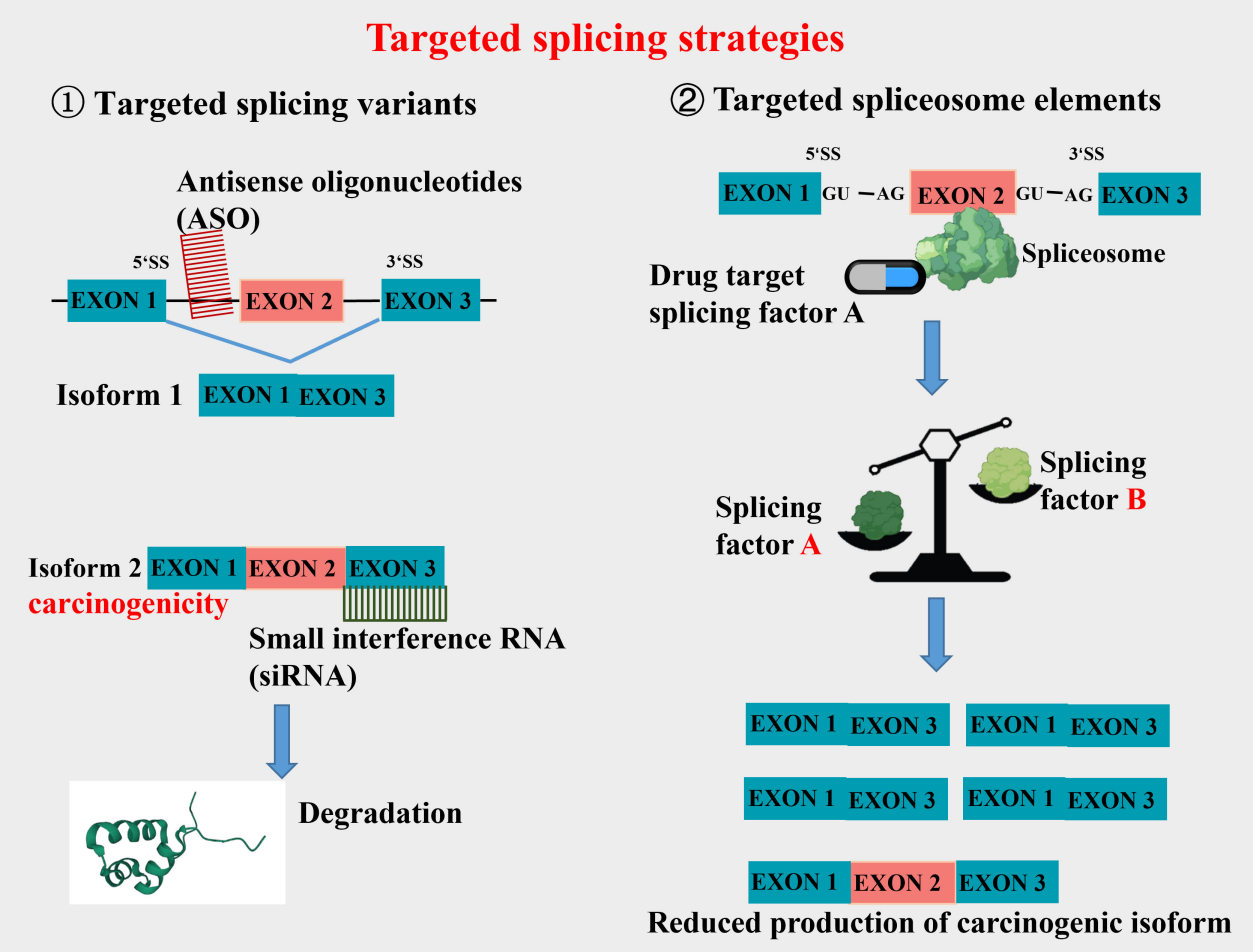
Schematic mechanisms of targeted splicing strategies. Strategies targeting splicing variants include Antisense oligonucleotides (ASO) and Small interfering RNA (siRNA). ASO can interact with specific exon or intron sequences of target mRNA and alter its splicing, thereby affecting the expression and function of the target gene. siRNA degrades targeted mRNA to inhibit the expression of the targeted gene. Drugs targeting splicing factors can affect the expression balance of splicing factors in the spliceosome, thereby reducing the production of carcinogenic isoforms.

In this review, we summarize the current progress in targeted therapies for these splicing variants and some potential therapeutic approaches (shown in **Figure 3** and **Table 1**). However, although numerous splicing isoforms have been identified, many of them have not yet been ascertained whether they match appropriate target treatments. Thus, further research is needed to improve our understanding and develop effective targeted therapies. Since it is accepted that tumor-associated splice variants have promising applications in CRC diagnosis and prognosis, subsequent work should be twofold. First, we will study new tumor-associated splice variants by experimental data, especially for the study of different CRC phenotypes, which is crucial to future targeted therapeutic approaches. Second, we will extend targeted splicing therapies and explore how to manipulate splicing to make targeted CRC therapies more safely, effectively, and accurately. To conclude, this article mainly reviews abnormal splicing events and related tumor-specific splicing variants in CRC, providing insight into targeted splicing therapy in CRC.

**Table 1:** Current drugs targeted splicing in the treatment of CRC.

Drug name	Type	Target	Phase	Reference
4μ8c	Small molecules	IRE1α - XBP1s	Preclinical	(282)
Morpholino antisense oligonucleotides	ASO	CCND1a/1b	Preclinical	(67)
	siRNA	CCND1b	Preclinical	(289)
Tautomycetin	Small molecules	SRSF1 - MKNK2	Preclinical	(117)
SRPIN340	Small molecules	SRPK1/2 - MKNK2	Preclinical	
H3B-8800	Small molecules	SF3b	I	(72)
DBS1	Small molecules	SRPKs - VEGF	Preclinical	(290)
Sulfasalazine	xCT inhibitor	CD44	I	(291)
RO5429083	Antibody	CD44	I	(135)
HA oligomers	Small molecules	HA - CD44	Preclinical	
IM7 or KM201	Antibody	CD44	Preclinical	
PEP-1	Peptide	CD44	Preclinical	
shRNA or miRNA	Small molecules	CD44	Preclinical	(133)
α-CD44v6 mAb	Antibody	CD44	Preclinical	
SM08502	Small molecules	SRSF	I	(292)
OS2966	Antibody	β1-integrins	I	(148)
YM155	Small molecules	Survivin	II	(157)
LY2181308	ASO	Survivin	II	
SPC3042, EZN-3042	ASO	Survivin	I	
Survivin-2B80-88	Antigenic peptide	Survivin	I	(155)
ABT-263	Small molecules	BCL-2/BCL-X	II	(213)
Tocilizumab	Antibody	IL-6R	III	(234)
FD-895	antibiotic	SF3b	Preclinical	(71)
Spliceostatin A (SSA)	Small molecules	SF3b	Preclinical	
C-13	Small molecules	SYK	Preclinical	(199)
Merestinib	Small molecules	RON	I	(56)
Dabrafenib, Vemurafenib, Sorafenib, Pazopanib and Ponatinib	Small molecules	RIP3	III	(28)
Binimetinib and Palbociclib	Small molecules	KRAS	II	(50)
10058-F4	Small molecules	ITGA6	Preclinical	(109)
HOXB-AS3	lncRNA	PKM	Preclinical	(256)
Indacaterol	Small molecules	SRSF6 - ZO-1	Preclinical	(183)
GYS32661, MBQ-167	Small molecules	RAC1	Preclinical	(81)
Olamkicept	Fusion-protein	IL6/sIL-6R	II	(235)
Anti-VEGFR2 antibodies	Antibody	VEGFR2	Preclinical	(229)
	siRNAs	VEGFR2	Preclinical	
Sunitinib, Pazopanib	Small molecules	VEGFR2	I	
Morpholino antisense oligonucleotides	ASO	VEGFR2	Preclinical	
ST2146	Antibody	TNC	Preclinical	(151)

## Author contributions

YZ and GZ collected the data and wrote the manuscript. CH designed and supervised the study. ML supervised the study. All authors have read and approved the final manuscript. All authors contributed to the article and approved the submitted version.

## References

[1] Siegel RL Miller KD Wagle NS Jemal A. Cancer statistics, 2023. CA Cancer J Clin (2023) 73(1):17–48. doi: 10.3322/caac.21763 36633525

[2] Jiang L Ping L Yan H Yang X He Q Xu Z. Cardiovascular toxicity induced by anti-VEGF/VEGFR agents: a special focus on definitions, diagnoses, mechanisms and management. Expert Opin Drug Metab Toxicol (2020) 16(9):823–35. doi: 10.1080/17425255.2020.1787986 32597258

[3] Wang E Aifantis I. RNA splicing and cancer. Trends Cancer (2020) 6(8):631–44. doi: 10.1016/j.trecan.2020.04.011 32434734

[4] Chow LT Gelinas RE Broker TR Roberts RJ. An amazing sequence arrangement at the 5’ ends of adenovirus 2 messenger RNA. Cell (1977) 12(1):1–8. doi: 10.1016/0092-8674(77)90180-5 902310

[5] Klessig DF. Two adenovirus mRNAs have a common 5’ terminal leader sequence encoded at least 10 kb upstream from their main coding regions. Cell (1977) 12(1):9–21. doi: 10.1016/0092-8674(77)90181-7 902321

[6] Liu Q Fang L Wu C. Alternative splicing and isoforms: from mechanisms to diseases. Genes (Basel) (2022) 13(3):401. doi: 10.3390/genes13030401 35327956 PMC8951537

[7] Miura K Fujibuchi W Unno M. Splice isoforms as therapeutic targets for colorectal cancer. Carcinogenesis (2012) 33(12):2311–9. doi: 10.1093/carcin/bgs347 23118106

[8] Chen L Tovar-Corona JM Urrutia AO. Increased levels of noisy splicing in cancers, but not for oncogene-derived transcripts. Hum Mol Genet (2011) 20(22):4422–9. doi: 10.1093/hmg/ddr370 PMC3196890 21862452

[9] Group PTC Calabrese C Davidson NR Demircioglu D Fonseca NA He Y. Genomic basis for RNA alterations in cancer. Nature (2020) 578(7793):129–36. doi: 10.1038/s41586-020-1970-0 PMC7054216 32025019

[10] Tian J Wang Z Mei S Yang N Yang Y Ke J. CancerSplicingQTL: a database for genome-wide identification of splicing QTLs in human cancer. Nucleic Acids Res (2019) 47(D1):D909–D16. doi: 10.1093/nar/gky954 PMC6324030 30329095

[11] He C Li A Lai Q Ding J Yan Q Liu S. The DDX39B/FUT3/TGFbetaR-I axis promotes tumor metastasis and EMT in colorectal cancer. Cell Death Dis (2021) 12(1):74. doi: 10.1038/s41419-020-03360-6 33436563 PMC7803960

[12] Bonnal SC Lopez-Oreja I Valcarcel J. Roles and mechanisms of alternative splicing in cancer - implications for care. Nat Rev Clin Oncol (2020) 17(8):457–74. doi: 10.1038/s41571-020-0350-x 32303702

[13] Dvinge H Bradley RK. Widespread intron retention diversifies most cancer transcriptomes. Genome Med (2015) 7(1):45. doi: 10.1186/s13073-015-0168-9 26113877 PMC4480902

[14] Capon DJ Seeburg PH McGrath JP Hayflick JS Edman U Levinson AD. Activation of Ki-ras2 gene in human colon and lung carcinomas by two different point mutations. Nature (1983) 304(5926):507–13. doi: 10.1038/304507a0 6308467

[15] Sciarrillo R Wojtuszkiewicz A Assaraf YG Jansen G Kaspers GJL Giovannetti E. The role of alternative splicing in cancer: From oncogenesis to drug resistance. Drug Resist Update (2020) 53:100728. doi: 10.1016/j.drup.2020.100728 33070093

[16] Marima R Francies FZ Hull R Molefi T Oyomno M Khanyile R. MicroRNA and alternative mRNA splicing events in cancer drug response/resistance: potent therapeutic targets. Biomedicines (2021) 9(12):1818. doi: 10.3390/biomedicines9121818 34944633 PMC8698559

[17] Lee SC Abdel-Wahab O. Therapeutic targeting of splicing in cancer. Nat Med (2016) 22(9):976–86. doi: 10.1038/nm.4165 PMC5644489 27603132

[18] Wang J Wang C Li L Yang L Wang S Ning X. Alternative splicing: An important regulatory mechanism in colorectal carcinoma. Mol Carcinog (2021) 60(4):279–93. doi: 10.1002/mc.23291 33629774

[19] Kole R Krainer AR Altman S. RNA therapeutics: beyond RNA interference and antisense oligonucleotides. Nat Rev Drug Discovery (2012) 11(2):125–40. doi: 10.1038/nrd3625 PMC4743652 22262036

[20] Cirak S Arechavala-Gomeza V Guglieri M Feng L Torelli S Anthony K. Exon skipping and dystrophin restoration in patients with Duchenne muscular dystrophy after systemic phosphorodiamidate morpholino oligomer treatment: an open-label, phase 2, dose-escalation study. Lancet (2011) 378(9791):595–605. doi: 10.1016/S0140-6736(11)60756-3 21784508 PMC3156980

[21] Zanetta C Nizzardo M Simone C Monguzzi E Bresolin N Comi GP. Molecular therapeutic strategies for spinal muscular atrophies: current and future clinical trials. Clin Ther (2014) 36(1):128–40. doi: 10.1016/j.clinthera.2013.11.006 24360800

[22] Larrayoz M Blakemore SJ Dobson RC Blunt MD Rose-Zerilli MJ Walewska R. The SF3B1 inhibitor spliceostatin A (SSA) elicits apoptosis in chronic lymphocytic leukaemia cells through downregulation of Mcl-1. Leukemia (2016) 30(2):351–60. doi: 10.1038/leu.2015.286 26488112

[23] Sciarrillo R Wojtuszkiewicz A Kooi IE Leon LG Sonneveld E Kuiper RP. Glucocorticoid resistant pediatric acute lymphoblastic leukemia samples display altered splicing profile and vulnerability to spliceosome modulation. Cancers (Basel) (2020) 12(3):723. doi: 10.3390/cancers12030723 32204435 PMC7140081

[24] Sun X Lee J Navas T Baldwin DT Stewart TA Dixit VM. RIP3, a novel apoptosis-inducing kinase. J Biol Chem (1999) 274(24):16871–5. doi: 10.1074/jbc.274.24.16871 10358032

[25] Liu ZY Zheng M Li YM Fan XY Wang JC Li ZC. RIP3 promotes colitis-associated colorectal cancer by controlling tumor cell proliferation and CXCL1-induced immune suppression. Theranostics (2019) 9(12):3659–73. doi: 10.7150/thno.32126 PMC6587173 31281505

[26] Liu S Joshi K Denning MF Zhang J. RIPK3 signaling and its role in the pathogenesis of cancers. Cell Mol Life Sci (2021) 78(23):7199–217. doi: 10.1007/s00018-021-03947-y PMC9044760 34654937

[27] Yang Y Hu W Feng S Ma J Wu M. RIP3 beta and RIP3 gamma, two novel splice variants of receptor-interacting protein 3 (RIP3), downregulate RIP3-induced apoptosis. Biochem Biophys Res Commun (2005) 332(1):181–7. doi: 10.1016/j.bbrc.2005.04.114 15896315

[28] Fulda S. Repurposing anticancer drugs for targeting necroptosis. Cell Cycle (2018) 17(7):829–32. doi: 10.1080/15384101.2018.1442626 PMC6056208 29464983

[29] Akiyama T. [The APC gene]. Nihon Rinsho (1996) 54(4):955–9. 8920656

[30] Schwarzova L Stekrova J Florianova M Novotny A Schneiderova M Lnenicka P. Novel mutations of the APC gene and genetic consequences of splicing mutations in the Czech FAP families. Fam Cancer (2013) 12(1):35–42. doi: 10.1007/s10689-012-9569-8 22987206

[31] Carson DJ Santoro IM Groden J. Isoforms of the APC tumor suppressor and their ability to inhibit cell growth and tumorigenicity. Oncogene (2004) 23(42):7144–8. doi: 10.1038/sj.onc.1207954 15273719

[32] Vaysse C Philippe C Martineau Y Quelen C Hieblot C Renaud C. Key contribution of eIF4H-mediated translational control in tumor promotion. Oncotarget (2015) 6(37):39924–40. doi: 10.18632/oncotarget.5442 PMC4741870 26498689

[33] Martindale DW Wilson MD Wang D Burke RD Chen X Duronio V. Comparative genomic sequence analysis of the Williams syndrome region (LIMK1-RFC2) of human chromosome 7q11.23. Mamm Genome (2000) 11(10):890–8. doi: 10.1007/s003350010166 11003705

[34] Wu D Matsushita K Matsubara H Nomura F Tomonaga T. An alternative splicing isoform of eukaryotic initiation factor 4H promotes tumorigenesis in *vivo* and is a potential therapeutic target for human cancer. Int J Cancer (2011) 128(5):1018–30. doi: 10.1002/ijc.25419 20473909

[35] Wu LC Wang ZW Tsan JT Spillman MA Phung A Xu XL. Identification of a RING protein that can interact in *vivo* with the BRCA1 gene product. Nat Genet (1996) 14(4):430–40. doi: 10.1038/ng1296-430 8944023

[36] Garcia-Patino E Gomendio B Lleonart M Silva JM Garcia JM Provencio M. Loss of heterozygosity in the region including the BRCA1 gene on 17q in colon cancer. Cancer Genet Cytogenet (1998) 104(2):119–23. doi: 10.1016/s0165-4608(97)00460-3 9666805

[37] Ford D Easton DF Bishop DT Narod SA Goldgar DE. Risks of cancer in BRCA1-mutation carriers. Breast Cancer Linkage Consortium. Lancet (1994) 343(8899):692–5. doi: 10.1016/s0140-6736(94)91578-4 7907678

[38] Brose MS Rebbeck TR Calzone KA Stopfer JE Nathanson KL Weber BL. Cancer risk estimates for BRCA1 mutation carriers identified in a risk evaluation program. J Natl Cancer Inst (2002) 94(18):1365–72. doi: 10.1093/jnci/94.18.1365 12237282

[39] Lin KM Ternent CA Adams DR Thorson AG Blatchford GJ Christensen MA. Colorectal cancer in hereditary breast cancer kindreds. Dis Colon Rectum (1999) 42(8):1041–5. doi: 10.1007/BF02236700 10458128

[40] Suchy J Cybulski C Gorski B Huzarski T Byrski T Debniak T. BRCA1 mutations and colorectal cancer in Poland. Fam Cancer (2010) 9(4):541–4. doi: 10.1007/s10689-010-9378-x 20862552

[41] Fabbro M Rodriguez JA Baer R Henderson BR. BARD1 induces BRCA1 intranuclear foci formation by increasing RING-dependent BRCA1 nuclear import and inhibiting BRCA1 nuclear export. J Biol Chem (2002) 277(24):21315–24. doi: 10.1074/jbc.M200769200 11925436

[42] Zhang YQ Pilyugin M Kuester D Leoni VP Li L Casula G. Expression of oncogenic BARD1 isoforms affects colon cancer progression and correlates with clinical outcome. Br J Cancer (2012) 107(4):675–83. doi: 10.1038/bjc.2012.297 PMC3419952 22814582

[43] Cimmino F Formicola D Capasso M. Dualistic role of BARD1 in cancer. Genes (Basel) (2017) 8(12):375. doi: 10.3390/genes8120375 29292755 PMC5748693

[44] Laszlo L Kurilla A Takacs T Kudlik G Koprivanacz K Buday L. Recent updates on the significance of KRAS mutations in colorectal cancer biology. Cells (2021) 10(3):667. doi: 10.3390/cells10030667 33802849 PMC8002639

[45] Prior IA Lewis PD Mattos C. A comprehensive survey of Ras mutations in cancer. Cancer Res (2012) 72(10):2457–67. doi: 10.1158/0008-5472.CAN-11-2612 PMC3354961 22589270

[46] Moore AR Rosenberg SC McCormick F Malek S. RAS-targeted therapies: is the undruggable drugged? Nat Rev Drug Discovery (2020) 19(8):533–52. doi: 10.1038/s41573-020-0068-6 PMC7809886 32528145

[47] Ahearn IM Haigis K Bar-Sagi D Philips MR. Regulating the regulator: post-translational modification of RAS. Nat Rev Mol Cell Biol (2011) 13(1):39–51. doi: 10.1038/nrm3255 22189424 PMC3879958

[48] Nuevo-Tapioles C Philips MR. The role of KRAS splice variants in cancer biology. Front Cell Dev Biol (2022) 10:1033348. doi: 10.3389/fcell.2022.1033348 36393833 PMC9663995

[49] Amendola CR Mahaffey JP Parker SJ Ahearn IM Chen WC Zhou M. KRAS4A directly regulates hexokinase 1. Nature (2019) 576(7787):482–6. doi: 10.1038/s41586-019-1832-9 PMC6923592 31827279

[50] Sorokin AV Kanikarla Marie P Bitner L Syed M Woods M Manyam G. Targeting RAS mutant colorectal cancer with dual inhibition of MEK and CDK4/6. Cancer Res (2022) 82(18):3335–44. doi: 10.1158/0008-5472.CAN-22-0198 PMC9478530 35913398

[51] Mayer S Hirschfeld M Jaeger M Pies S Iborra S Erbes T. RON alternative splicing regulation in primary ovarian cancer. Oncol Rep (2015) 34(1):423–30. doi: 10.3892/or.2015.3995 25997828

[52] Collesi C Santoro MM Gaudino G Comoglio PM. A splicing variant of the RON transcript induces constitutive tyrosine kinase activity and an invasive phenotype. Mol Cell Biol (1996) 16(10):5518–26. doi: 10.1128/MCB.16.10.5518 PMC231551 8816464

[53] Zhou YQ He C Chen YQ Wang D Wang MH. Altered expression of the RON receptor tyrosine kinase in primary human colorectal adenocarcinomas: generation of different splicing RON variants and their oncogenic potential. Oncogene (2003) 22(2):186–97. doi: 10.1038/sj.onc.1206075 12527888

[54] Wang MH Lao WF Wang D Luo YL Yao HP. Blocking tumorigenic activities of colorectal cancer cells by a splicing RON receptor variant defective in the tyrosine kinase domain. Cancer Biol Ther (2007) 6(7):1121–9. doi: 10.4161/cbt.6.7.4337 17611409

[55] Ghigna C Giordano S Shen H Benvenuto F Castiglioni F Comoglio PM. Cell motility is controlled by SF2/ASF through alternative splicing of the Ron protooncogene. Mol Cell (2005) 20(6):881–90. doi: 10.1016/j.molcel.2005.10.026 16364913

[56] He AR Cohen RB Denlinger CS Sama A Birnbaum A Hwang J. First-in-human phase I study of merestinib, an oral multikinase inhibitor, in patients with advanced cancer. Oncologist (2019) 24(9):e930–e42. doi: 10.1634/theoncologist.2018-0411 PMC6738318 30833489

[57] Montalto FI De Amicis F. Cyclin D1 in cancer: A molecular connection for cell cycle control, adhesion and invasion in tumor and stroma. Cells (2020) 9(12):2648. doi: 10.3390/cells9122648 33317149 PMC7763888

[58] Yan H Jiang F Yang J. Association of beta-catenin, APC, SMAD3/4, tp53, and cyclin D1 genes in colorectal cancer: A systematic review and meta-analysis. Genet Res (Camb) (2022) 2022:5338956. doi: 10.1155/2022/5338956 36072013 PMC9402361

[59] Bahnassy AA Zekri AR El-Houssini S El-Shehaby AM Mahmoud MR Abdallah S. Cyclin A and cyclin D1 as significant prognostic markers in colorectal cancer patients. BMC Gastroenterol (2004) 4:22. doi: 10.1186/1471-230X-4-22 15385053 PMC524166

[60] Yang Y Wang F Shi C Zou Y Qin H Ma Y. Cyclin D1 G870A polymorphism contributes to colorectal cancer susceptibility: evidence from a systematic review of 22 case-control studies. PLoS One (2012) 7(5):e36813. doi: 10.1371/journal.pone.0036813 22606291 PMC3350479

[61] Garcia-Aguilar J Chen Z Smith DD Li W Madoff RD Cataldo P. Identification of a biomarker profile associated with resistance to neoadjuvant chemoradiation therapy in rectal cancer. Ann Surg (2011) 254(3):486–92. doi: 10.1097/SLA.0b013e31822b8cfa PMC3202983 21865946

[62] El Menshawy N El Marghany AB Sarhan MM Aladle DA. Cyclin D1 G870A polymorphism: relation to the risk of ALL development, prognosis impact, and methotrexate cytotoxicity. Asian Pac J Cancer Prev (2020) 21(10):2941–7. doi: 10.31557/APJCP.2020.21.10.2941 PMC7798150 33112552

[63] Howe D Lynas C. The cyclin D1 alternative transcripts [a] and [b] are expressed in normal and Malignant lymphocytes and their relative levels are influenced by the polymorphism at codon 241. Haematologica (2001) 86(6):563–9. 11418364

[64] Betticher DC Thatcher N Altermatt HJ Hoban P Ryder WD Heighway J. Alternate splicing produces a novel cyclin D1 transcript. Oncogene (1995) 11(5):1005–11. 7675441

[65] Wu FH Luo LQ Liu Y Zhan QX Luo C Luo J. Cyclin D1b splice variant promotes alphavbeta3-mediated adhesion and invasive migration of breast cancer cells. Cancer Lett (2014) 355(1):159–67. doi: 10.1016/j.canlet.2014.08.044 25193465

[66] Kim CJ Tambe Y Mukaisho KI Sugihara H Kawauchi A Inoue H. Akt-dependent activation of Erk by cyclin D1b contributes to cell invasiveness and tumorigenicity. Oncol Lett (2016) 12(6):4850–6. doi: 10.3892/ol.2016.5286 PMC5228369 28105192

[67] Wang J Su W Zhang T Zhang S Lei H Ma F. Aberrant Cyclin D1 splicing in cancer: from molecular mechanism to therapeutic modulation. Cell Death Dis (2023) 14(4):244. doi: 10.1038/s41419-023-05763-7 37024471 PMC10079974

[68] Matsushita K Tomonaga T Shimada H Shioya A Higashi M Matsubara H. An essential role of alternative splicing of c-myc suppressor FUSE-binding protein-interacting repressor in carcinogenesis. Cancer Res (2006) 66(3):1409–17. doi: 10.1158/0008-5472.CAN-04-4459 16452196

[69] Kajiwara T Matsushita K Itoga S Tamura M Tanaka N Tomonaga T. SAP155-mediated c-myc suppressor far-upstream element-binding protein-interacting repressor splicing variants are activated in colon cancer tissues. Cancer Sci (2013) 104(2):149–56. doi: 10.1111/cas.12058 PMC7657139 23113893

[70] Matsushita K Kajiwara T Tamura M Satoh M Tanaka N Tomonaga T. SAP155-mediated splicing of FUSE-binding protein-interacting repressor serves as a molecular switch for c-myc gene expression. Mol Cancer Res (2012) 10(6):787–99. doi: 10.1158/1541-7786.MCR-11-0462 PMC7469880 22496461

[71] Martinez-Montiel N Rosas-Murrieta NH Anaya Ruiz M Monjaraz-Guzman E Martinez-Contreras R. Alternative splicing as a target for cancer treatment. Int J Mol Sci (2018) 19(2):545. doi: 10.3390/ijms19020545 29439487 PMC5855767

[72] Steensma DP Wermke M Klimek VM Greenberg PL Font P Komrokji RS. Phase I First-in-Human Dose Escalation Study of the oral SF3B1 modulator H3B-8800 in myeloid neoplasms. Leukemia (2021) 35(12):3542–50. doi: 10.1038/s41375-021-01328-9 PMC8632688 34172893

[73] Svensmark JH Brakebusch C. Rho GTPases in cancer: friend or foe? Oncogene (2019) 38(50):7447–56. doi: 10.1038/s41388-019-0963-7 31427738

[74] Lee K Chen QK Lui C Cichon MA Radisky DC Nelson CM. Matrix compliance regulates Rac1b localization, NADPH oxidase assembly, and epithelial-mesenchymal transition. Mol Biol Cell (2012) 23(20):4097–108. doi: 10.1091/mbc.E12-02-0166 PMC3469523 22918955

[75] Aljagthmi AA Hill NT Cooke M Kazanietz MG Abba MC Long W. DeltaNp63alpha suppresses cells invasion by downregulating PKCgamma/Rac1 signaling through miR-320a. Cell Death Dis (2019) 10(9):680. doi: 10.1038/s41419-019-1921-6 31515469 PMC6742631

[76] Goncalves V Henriques AF Pereira JF Neves Costa A Moyer MP Moita LF. Phosphorylation of SRSF1 by SRPK1 regulates alternative splicing of tumor-related Rac1b in colorectal cells. RNA (2014) 20(4):474–82. doi: 10.1261/rna.041376.113 PMC3964909 24550521

[77] Wang F Fu X Chen P Wu P Fan X Li N. SPSB1-mediated HnRNP A1 ubiquitylation regulates alternative splicing and cell migration in EGF signaling. Cell Res (2017) 27(4):540–58. doi: 10.1038/cr.2017.7 PMC5385621 28084329

[78] Goncalves V Matos P Jordan P. Antagonistic SR proteins regulate alternative splicing of tumor-related Rac1b downstream of the PI3-kinase and Wnt pathways. Hum Mol Genet (2009) 18(19):3696–707. doi: 10.1093/hmg/ddp317 19602482

[79] Gudino V Pohl SO Billard CV Cammareri P Bolado A Aitken S. RAC1B modulates intestinal tumourigenesis *via* modulation of WNT and EGFR signalling pathways. Nat Commun (2021) 12(1):2335. doi: 10.1038/s41467-021-22531-3 33879799 PMC8058071

[80] Kotelevets L Chastre E. Rac1 signaling: from intestinal homeostasis to colorectal cancer metastasis. Cancers (Basel) (2020) 12(3):665. doi: 10.3390/cancers12030665 32178475 PMC7140047

[81] Bailly C Beignet J LoIrand G Sauzeau V. Rac1 as a therapeutic anticancer target: Promises and limitations. Biochem Pharmacol (2022) 203:115180. doi: 10.1016/j.bcp.2022.115180 35853497

[82] Alonso-Espinaco V Cuatrecasas M Alonso V Escudero P Marmol M Horndler C. RAC1b overexpression correlates with poor prognosis in KRAS/BRAF WT metastatic colorectal cancer patients treated with first-line FOLFOX/XELOX chemotherapy. Eur J Cancer (2014) 50(11):1973–81. doi: 10.1016/j.ejca.2014.04.019 24833563

[83] Goka ET Chaturvedi P Lopez DTM Garza A Lippman ME. RAC1b overexpression confers resistance to chemotherapy treatment in colorectal cancer. Mol Cancer Ther (2019) 18(5):957–68. doi: 10.1158/1535-7163.MCT-18-0955 30926638

[84] Abdel-Samad R Zalzali H Rammah C Giraud J Naudin C Dupasquier S. MiniSOX9, a dominant-negative variant in colon cancer cells. Oncogene (2011) 30(22):2493–503. doi: 10.1038/onc.2010.621 21297661

[85] Thorsen K Mansilla F Schepeler T Oster B Rasmussen MH Dyrskjot L. Alternative splicing of SLC39A14 in colorectal cancer is regulated by the Wnt pathway. Mol Cell Proteomics (2011) 10(1):M110. doi: 10.1074/mcp.M110.002998 PMC3013455 20938052

[86] Sveen A Bakken AC Agesen TH Lind GE Nesbakken A Nordgard O. The exon-level biomarker SLC39A14 has organ-confined cancer-specificity in colorectal cancer. Int J Cancer (2012) 131(6):1479–85. doi: 10.1002/ijc.27399 22173985

[87] Smeby J Sveen A Eilertsen IA Danielsen SA Hoff AM Eide PW. Transcriptional and functional consequences of TP53 splice mutations in colorectal cancer. Oncogenesis (2019) 8(6):35. doi: 10.1038/s41389-019-0141-3 31092812 PMC6520361

[88] Shirole NH Pal D Kastenhuber ER Senturk S Boroda J Pisterzi P. TP53 exon-6 truncating mutations produce separation of function isoforms with pro-tumorigenic functions. Elife (2016) 5:e17929. doi: 10.7554/eLife.17929 27759562 PMC5092050

[89] Senturk S Yao Z Camiolo M Stiles B Rathod T Walsh AM. p53Psi is a transcriptionally inactive p53 isoform able to reprogram cells toward a metastatic-like state. Proc Natl Acad Sci U.S.A. (2014) 111(32):E3287–96. doi: 10.1073/pnas.1321640111 PMC4136628 25074920

[90] Montero-Calle A Garranzo-Asensio M Torrente-Rodriguez RM Ruiz-Valdepenas Montiel V Poves C Dziakova J. p53 and p63 proteoforms derived from alternative splicing possess differential seroreactivity in colorectal cancer with distinct diagnostic ability from the canonical proteins. Cancers (Basel) (2023) 15(7):2102. doi: 10.3390/cancers15072102 37046764 PMC10092954

[91] Zhou X Li X Cheng Y Wu W Xie Z Xi Q. BCLAF1 and its splicing regulator SRSF10 regulate the tumorigenic potential of colon cancer cells. Nat Commun (2014) 5:4581. doi: 10.1038/ncomms5581 25091051

[92] Pibouin L Villaudy J Ferbus D Muleris M Prosperi MT Remvikos Y. Cloning of the mRNA of overexpression in colon carcinoma-1: a sequence overexpressed in a subset of colon carcinomas. Cancer Genet Cytogenet (2002) 133(1):55–60. doi: 10.1016/s0165-4608(01)00634-3 11890990

[93] Najafi H Soltani BM Dokanehiifard S Nasiri S Mowla SJ. Alternative splicing of the OCC-1 gene generates three splice variants and a novel exonic microRNA, which regulate the Wnt signaling pathway. RNA (2017) 23(1):70–85. doi: 10.1261/rna.056317.116 27986894 PMC5159651

[94] Yusuf D Butland SL Swanson MI Bolotin E Ticoll A Cheung WA. The transcription factor encyclopedia. Genome Biol (2012) 13(3):R24. doi: 10.1186/gb-2012-13-3-r24 22458515 PMC3439975

[95] Vuong LM Chellappa K Dhahbi JM Deans JR Fang B Bolotin E. Differential effects of hepatocyte nuclear factor 4alpha isoforms on tumor growth and T-cell factor 4/AP-1 interactions in human colorectal cancer cells. Mol Cell Biol (2015) 35(20):3471–90. doi: 10.1128/MCB.00030-15 PMC4573706 26240283

[96] Changolkar LN Pehrson JR. macroH2A1 histone variants are depleted on active genes but concentrated on the inactive X chromosome. Mol Cell Biol (2006) 26(12):4410–20. doi: 10.1128/MCB.02258-05 PMC1489112 16738309

[97] Kim SP Srivatsan SN Chavez M Shirai CL White BS Ahmed T. Mutant U2AF1-induced alternative splicing of H2afy (macroH2A1) regulates B-lymphopoiesis in mice. Cell Rep (2021) 36(9):109626. doi: 10.1016/j.celrep.2021.109626 34469727 PMC8454217

[98] Novikov L Park JW Chen H Klerman H Jalloh AS Gamble MJ. QKI-mediated alternative splicing of the histone variant MacroH2A1 regulates cancer cell proliferation. Mol Cell Biol (2011) 31(20):4244–55. doi: 10.1128/MCB.05244-11 PMC3187283 21844227

[99] Fei DL Zhen T Durham B Ferrarone J Zhang T Garrett L. Impaired hematopoiesis and leukemia development in mice with a conditional knock-in allele of a mutant splicing factor gene U2af1. Proc Natl Acad Sci U S A (2018) 115(44):E10437–E46. doi: 10.1073/pnas.1812669115 PMC6217397 30322915

[100] Ilagan JO Ramakrishnan A Hayes B Murphy ME Zebari AS Bradley P. U2AF1 mutations alter splice site recognition in hematological Malignancies. Genome Res (2015) 25(1):14–26. doi: 10.1101/gr.181016.114 25267526 PMC4317169

[101] Yip BH Steeples V Repapi E Armstrong RN Llorian M Roy S. The U2AF1S34F mutation induces lineage-specific splicing alterations in myelodysplastic syndromes. J Clin Invest (2017) 127(9):3557. doi: 10.1172/JCI96202 PMC5669537 28862641

[102] Lu SC Mato JM. S-Adenosylmethionine in cell growth, apoptosis and liver cancer. J Gastroenterol Hepatol (2008) 23 Suppl 1(Suppl 1):S73–7. doi: 10.1111/j.1440-1746.2007.05289.x PMC2408691 18336669

[103] Yang H Ara AI Magilnick N Xia M Ramani K Chen H. Expression pattern, regulation, and functions of methionine adenosyltransferase 2beta splicing variants in hepatoma cells. Gastroenterology (2008) 134(1):281–91. doi: 10.1053/j.gastro.2007.10.027 PMC2409110 18045590

[104] Xia M Chen Y Wang LC Zandi E Yang H Bemanian S. Novel function and intracellular localization of methionine adenosyltransferase 2beta splicing variants. J Biol Chem (2010) 285(26):20015–21. doi: 10.1074/jbc.M109.094821 PMC2888413 20421296

[105] Margadant C Monsuur HN Norman JC Sonnenberg A. Mechanisms of integrin activation and trafficking. Curr Opin Cell Biol (2011) 23(5):607–14. doi: 10.1016/j.ceb.2011.08.005 21924601

[106] Hogervorst F Admiraal LG Niessen C Kuikman I Janssen H Daams H. Biochemical characterization and tissue distribution of the A and B variants of the integrin alpha 6 subunit. J Cell Biol (1993) 121(1):179–91. doi: 10.1083/jcb.121.1.179 PMC2119779 7681434

[107] Groulx JF Giroux V Beausejour M Boudjadi S Basora N Carrier JC. Integrin alpha6A splice variant regulates proliferation and the Wnt/beta-catenin pathway in human colorectal cancer cells. Carcinogenesis (2014) 35(6):1217–27. doi: 10.1093/carcin/bgu006 PMC4043246 24403311

[108] van der Flier LG Clevers H. Stem cells, self-renewal, and differentiation in the intestinal epithelium. Annu Rev Physiol (2009) 71:241–60. doi: 10.1146/annurev.physiol.010908.163145 18808327

[109] Groulx JF Boudjadi S Beaulieu JF. MYC regulates alpha6 integrin subunit expression and splicing under its pro-proliferative ITGA6A form in colorectal cancer cells. Cancers (Basel) (2018) 10(2):42. doi: 10.3390/cancers10020042 29401653 PMC5836074

[110] Bao X Huang Y Xu W Xiong G. Functions and clinical significance of UPF3a expression in human colorectal cancer. Cancer Manag Res (2020) 12:4271–81. doi: 10.2147/CMAR.S244486 PMC7292372 32606924

[111] Shum EY Jones SH Shao A Chousal JN Krause MD Chan WK. The antagonistic gene paralogs Upf3a and Upf3b govern nonsense-mediated RNA decay. Cell (2016) 165(2):382–95. doi: 10.1016/j.cell.2016.02.046 PMC4826573 27040500

[112] Wang Q Wang Y Liu Y Zhang C Luo Y Guo R. U2-related proteins CHERP and SR140 contribute to colorectal tumorigenesis *via* alternative splicing regulation. Int J Cancer (2019) 145(10):2728–39. doi: 10.1002/ijc.32331 30977118

[113] Xu W Ou W Feng Y Xu Q Yang Y Cui L. Genetic compensation response could exist in colorectal cancer: UPF3A upregulates the oncogenic homologue gene SRSF3 expression corresponding to SRSF6 to promote colorectal cancer metastasis. J Gastroenterol Hepatol (2023) 38(4):634–47. doi: 10.1111/jgh.16152 36807382

[114] Yang X Zhong W Cao R. Phosphorylation of the mRNA cap-binding protein eIF4E and cancer. Cell Signal (2020) 73:109689. doi: 10.1016/j.cellsig.2020.109689 32535199 PMC8049097

[115] Scheper GC Parra JL Wilson M Van Kollenburg B Vertegaal AC Han ZG. The N and C termini of the splice variants of the human mitogen-activated protein kinase-interacting kinase Mnk2 determine activity and localization. Mol Cell Biol (2003) 23(16):5692–705. doi: 10.1128/MCB.23.16.5692-5705.2003 PMC166352 12897141

[116] Maimon A Mogilevsky M Shilo A Golan-Gerstl R Obiedat A Ben-Hur V. Mnk2 alternative splicing modulates the p38-MAPK pathway and impacts Ras-induced transformation. Cell Rep (2014) 7(2):501–13. doi: 10.1016/j.celrep.2014.03.041 24726367

[117] Liu H Gong Z Li K Zhang Q Xu Z Xu Y. SRPK1/2 and PP1alpha exert opposite functions by modulating SRSF1-guided MKNK2 alternative splicing in colon adenocarcinoma. J Exp Clin Cancer Res (2021) 40(1):75. doi: 10.1186/s13046-021-01877-y 33602301 PMC7893936

[118] Zhang H Duan HO Kirley SD Zukerberg LR Wu CL. Aberrant splicing of cables gene, a CDK regulator, in human cancers. Cancer Biol Ther (2005) 4(11):1211–5. doi: 10.4161/cbt.4.11.2085 16177568

[119] Ikeuchi K Marusawa H Fujiwara M Matsumoto Y Endo Y Watanabe T. Attenuation of proteolysis-mediated cyclin E regulation by alternatively spliced Parkin in human colorectal cancers. Int J Cancer (2009) 125(9):2029–35. doi: 10.1002/ijc.24565 19585504

[120] Croft A Guo ST Sherwin S Farrelly M Yan XG Zhang XD. Functional identification of a novel transcript variant of INPP4B in human colon and breast cancer cells. Biochem Biophys Res Commun (2017) 485(1):47–53. doi: 10.1016/j.bbrc.2017.02.012 28189677

[121] Flodrops M Dujardin G Busson A Trouve P Ka C Simon B. TIMP1 intron 3 retention is a marker of colon cancer progression controlled by hnRNPA1. Mol Biol Rep (2020) 47(4):3031–40. doi: 10.1007/s11033-020-05375-w 32200451

[122] Klupp F Giese C Halama N Franz C Lasitschka F Warth A. E3 ubiquitin ligase Smurf2: a prognostic factor in microsatellite stable colorectal cancer. Cancer Manag Res (2019) 11:1795–803. doi: 10.2147/CMAR.S178111 PMC6391146 30863185

[123] Dornhoff H Becker C Wirtz S Strand D Tenzer S Rosfa S. A variant of Smurf2 protects mice against colitis-associated colon cancer by inducing transforming growth factor beta signaling. Gastroenterology (2012) 142(5):1183–94.e4. doi: 10.1053/j.gastro.2012.02.005 22333948

[124] Chen L Luo C Shen L Liu Y Wang Q Zhang C. SRSF1 prevents DNA damage and promotes Tumorigenesis through regulation of DBF4B pre-mRNA splicing. Cell Rep (2017) 21(12):3406–13. doi: 10.1016/j.celrep.2017.11.091 29262322

[125] Kajita K Kuwano Y Satake Y Kano S Kurokawa K Akaike Y. Ultraconserved region-containing Transformer 2beta4 controls senescence of colon cancer cells. Oncogenesis (2016) 5(4):e213. doi: 10.1038/oncsis.2016.18 27043659 PMC4848834

[126] Satake Y Kuwano Y Nishikawa T Fujita K Saijo S Itai M. Nucleolin facilitates nuclear retention of an ultraconserved region containing TRA2beta4 and accelerates colon cancer cell growth. Oncotarget (2018) 9(42):26817–33. doi: 10.18632/oncotarget.25510 PMC6003563 29928487

[127] Nishikawa T Kuwano Y Takahara Y Nishida K Rokutan K. HnRNPA1 interacts with G-quadruplex in the TRA2B promoter and stimulates its transcription in human colon cancer cells. Sci Rep (2019) 9(1):10276. doi: 10.1038/s41598-019-46659-x 31311954 PMC6635519

[128] Meteoglu I Meydan N Erkus M. Id-1: regulator of EGFR and VEGF and potential target for colorectal cancer therapy. J Exp Clin Cancer Res (2008) 27(1):69. doi: 10.1186/1756-9966-27-69 19014499 PMC2588562

[129] Manrique I Nguewa P Bleau AM Nistal-Villan E Lopez I Villalba M. The inhibitor of differentiation isoform Id1b, generated by alternative splicing, maintains cell quiescence and confers self-renewal and cancer stem cell-like properties. Cancer Lett (2015) 356(2 Pt B):899–909. doi: 10.1016/j.canlet.2014.10.035 25449776

[130] Muys BR Shrestha RL Anastasakis DG Pongor L Li XL Grammatikakis I. Matrin3 regulates mitotic spindle dynamics by controlling alternative splicing of CDC14B. Cell Rep (2023) 42(3):112260. doi: 10.1016/j.celrep.2023.112260 36924503 PMC10132239

[131] Prochazka L Tesarik R Turanek J. Regulation of alternative splicing of CD44 in cancer. Cell Signal (2014) 26(10):2234–9. doi: 10.1016/j.cellsig.2014.07.011 25025570

[132] Zeilstra J Joosten SP van Andel H Tolg C Berns A Snoek M. Stem cell CD44v isoforms promote intestinal cancer formation in Apc(min) mice downstream of Wnt signaling. Oncogene (2014) 33(5):665–70. doi: 10.1038/onc.2012.611 23318432

[133] Ma L Dong L Chang P. CD44v6 engages in colorectal cancer progression. Cell Death Dis (2019) 10(1):30. doi: 10.1038/s41419-018-1265-7 30631039 PMC6328617

[134] Orian-Rousseau V Ponta H. Perspectives of CD44 targeting therapies. Arch Toxicol (2015) 89(1):3–14. doi: 10.1007/s00204-014-1424-2 25472903

[135] Chen C Zhao S Karnad A Freeman JW. The biology and role of CD44 in cancer progression: therapeutic implications. J Hematol Oncol (2018) 11(1):64. doi: 10.1186/s13045-018-0605-5 29747682 PMC5946470

[136] Ejima R Suzuki H Tanaka T Asano T Kaneko MK Kato Y. Development of a novel anti-CD44 variant 6 monoclonal antibody C(44)Mab-9 for multiple applications against colorectal carcinomas. Int J Mol Sci (2023) 24(4):4007. doi: 10.3390/ijms24044007 36835416 PMC9965047

[137] Zhang LH Tian B Diao LR Xiong YY Tian SF Zhang BH. Dominant expression of 85-kDa form of cortactin in colorectal cancer. J Cancer Res Clin Oncol (2006) 132(2):113–20. doi: 10.1007/s00432-005-0046-8 PMC12161045 16261345

[138] van Rossum AG de Graaf JH Schuuring-Scholtes E Kluin PM Fan YX Zhan X. Alternative splicing of the actin binding domain of human cortactin affects cell migration. J Biol Chem (2003) 278(46):45672–9. doi: 10.1074/jbc.M306688200 12952985

[139] Wang ZN Liu D Yin B Ju WY Qiu HZ Xiao Y. High expression of PTBP1 promote invasion of colorectal cancer by alternative splicing of cortactin. Oncotarget (2017) 8(22):36185–202. doi: 10.18632/oncotarget.15873 PMC5482648 28404950

[140] Corsi JM Rouer E Girault JA Enslen H. Organization and post-transcriptional processing of focal adhesion kinase gene. BMC Genomics (2006) 7:198. doi: 10.1186/1471-2164-7-198 16889663 PMC1570463

[141] Burgaya F Toutant M Studler JM Costa A Le Bert M Gelman M. Alternatively spliced focal adhesion kinase in rat brain with increased autophosphorylation activity. J Biol Chem (1997) 272(45):28720–5. doi: 10.1074/jbc.272.45.28720 9353341

[142] Devaud C Tilkin-Mariame AF Vignolle-Vidoni A Souleres P Denadai-Souza A Rolland C. FAK alternative splice mRNA variants expression pattern in colorectal cancer. Int J Cancer (2019) 145(2):494–502. doi: 10.1002/ijc.32120 30628725 PMC6563491

[143] Midwood KS Hussenet T Langlois B Orend G. Advances in tenascin-C biology. Cell Mol Life Sci (2011) 68(19):3175–99. doi: 10.1007/s00018-011-0783-6 PMC3173650 21818551

[144] Giblin SP Midwood KS. Tenascin-C: Form versus function. Cell Adh Migr (2015) 9(1-2):48–82. doi: 10.4161/19336918.2014.987587 25482829 PMC4422809

[145] Saito Y Imazeki H Miura S Yoshimura T Okutsu H Harada Y. A peptide derived from tenascin-C induces beta1 integrin activation through syndecan-4. J Biol Chem (2007) 282(48):34929–37. doi: 10.1074/jbc.M705608200 17901052

[146] Tanaka R Seki Y Saito Y Kamiya S Fujita M Okutsu H. Tenascin-C-derived peptide TNIIIA2 highly enhances cell survival and platelet-derived growth factor (PDGF)-dependent cell proliferation through potentiated and sustained activation of integrin alpha5beta1. J Biol Chem (2014) 289(25):17699–708. doi: 10.1074/jbc.M113.546622 PMC4067204 24808173

[147] Dueck M Riedl S Hinz U Tandara A Mller P Herfarth C. Detection of tenascin-C isoforms in colorectal mucosa, ulcerative colitis, carcinomas and liver metastases. Int J Cancer (1999) 82(4):477–83. doi: 10.1002/(sici)1097-0215(19990812)82:4<477::Aid-ijc2>3.0.Co;2-5 10404058

[148] Nwagwu CD Immidisetti AV Bukanowska G Vogelbaum MA Carbonell AM. Convection-enhanced delivery of a first-in-class anti-beta1 integrin antibody for the treatment of high-grade glioma utilizing real-time imaging. Pharmaceutics (2020) 13(1):40. doi: 10.3390/pharmaceutics13010040 33396712 PMC7823464

[149] Suzuki H Sasada M Kamiya S Ito Y Watanabe H Okada Y. The promoting effect of the extracellular matrix peptide TNIIIA2 derived from tenascin-C in colon cancer cell infiltration. Int J Mol Sci (2017) 18(1):181. doi: 10.3390/ijms18010181 28106752 PMC5297813

[150] Fujita M Ito-Fujita Y Iyoda T Sasada M Okada Y Ishibashi K. Peptide TNIIIA2 Derived from Tenascin-C Contributes to Malignant Progression in Colitis-Associated Colorectal Cancer *via* beta1-Integrin Activation in Fibroblasts. Int J Mol Sci (2019) 20(11):2752. doi: 10.3390/ijms20112752 31195598 PMC6601010

[151] De Santis R Albertoni C Petronzelli F Campo S D’Alessio V Rosi A. Low and high tenascin-expressing tumors are efficiently targeted by ST2146 monoclonal antibody. Clin Cancer Res (2006) 12(7 Pt 1):2191–6. doi: 10.1158/1078-0432.CCR-05-2526 16609034

[152] Li F. Role of survivin and its splice variants in tumorigenesis. Br J Cancer (2005) 92(2):212–6. doi: 10.1038/sj.bjc.6602340 PMC2361850 15611788

[153] Ge QX Li YY Nie YQ Zuo WG Du YL. Expression of survivin and its four splice variants in colorectal cancer and its clinical significances. Med Oncol (2013) 30(2):535. doi: 10.1007/s12032-013-0535-6 23494669

[154] Noton EA Colnaghi R Tate S Starck C Carvalho A Ko Ferrigno P. Molecular analysis of survivin isoforms: evidence that alternatively spliced variants do not play a role in mitosis. J Biol Chem (2006) 281(2):1286–95. doi: 10.1074/jbc.M508773200 16291752

[155] Tanaka T Kitamura H Inoue R Nishida S Takahashi-Takaya A Kawami S. Potential survival benefit of anti-apoptosis protein: survivin-derived peptide vaccine with and without interferon alpha therapy for patients with advanced or recurrent urothelial cancer–results from phase I clinical trials. Clin Dev Immunol (2013) 2013:262967. doi: 10.1155/2013/262967 24363758 PMC3863714

[156] Kameshima H Tsuruma T Torigoe T Takahashi A Hirohashi Y Tamura Y. Immunogenic enhancement and clinical effect by type-I interferon of anti-apoptotic protein, survivin-derived peptide vaccine, in advanced colorectal cancer patients. Cancer Sci (2011) 102(6):1181–7. doi: 10.1111/j.1349-7006.2011.01918.x 21371173

[157] Martinez-Garcia D Manero-Ruperez N Quesada R Korrodi-Gregorio L Soto-Cerrato V. Therapeutic strategies involving survivin inhibition in cancer. Med Res Rev (2019) 39(3):887–909. doi: 10.1002/med.21547 30421440

[158] Kobayashi T Masaki T Nozaki E Sugiyama M Nagashima F Furuse J. Microarray analysis of gene expression at the tumor front of colon cancer. Anticancer Res (2015) 35(12):6577–81. 26637872

[159] Datta D Flaxenburg JA Laxmanan S Geehan C Grimm M Waaga-Gasser AM. Ras-induced modulation of CXCL10 and its receptor splice variant CXCR3-B in MDA-MB-435 and MCF-7 cells: relevance for the development of human breast cancer. Cancer Res (2006) 66(19):9509–18. doi: 10.1158/0008-5472.CAN-05-4345 17018607

[160] Bodnar RJ Yates CC Wells A. IP-10 blocks vascular endothelial growth factor-induced endothelial cell motility and tube formation *via* inhibition of calpain. Circ Res (2006) 98(5):617–25. doi: 10.1161/01.RES.0000209968.66606.10 PMC3826264 16484616

[161] Groom JR Luster AD. CXCR3 ligands: redundant, collaborative and antagonistic functions. Immunol Cell Biol (2011) 89(2):207–15. doi: 10.1038/icb.2010.158 PMC3863330 21221121

[162] Bai M Chen X Ba YI. CXCL10/CXCR3 overexpression as a biomarker of poor prognosis in patients with stage II colorectal cancer. Mol Clin Oncol (2016) 4(1):23–30. doi: 10.3892/mco.2015.665 26870351 PMC4726926

[163] Billottet C Quemener C Bikfalvi A. CXCR3, a double-edged sword in tumor progression and angiogenesis. Biochim Biophys Acta (2013) 1836(2):287–95. doi: 10.1016/j.bbcan.2013.08.002 23994549

[164] Ehlert JE Addison CA Burdick MD Kunkel SL Strieter RM. Identification and partial characterization of a variant of human CXCR3 generated by posttranscriptional exon skipping. J Immunol (2004) 173(10):6234–40. doi: 10.4049/jimmunol.173.10.6234 15528361

[165] Yang C Zheng W Du W. CXCR3A contributes to the invasion and metastasis of gastric cancer cells. Oncol Rep (2016) 36(3):1686–92. doi: 10.3892/or.2016.4953 27461521

[166] Wu Q Dhir R Wells A. Altered CXCR3 isoform expression regulates prostate cancer cell migration and invasion. Mol Cancer (2012) 11:3. doi: 10.1186/1476-4598-11-3 22236567 PMC3320557

[167] Nozaki E Kobayashi T Ohnishi H Ohtsuka K Masaki T Watanabe T. C-X-C motif receptor 3A enhances proliferation and invasiveness of colorectal cancer cells, and is mediated by C-X-C motif ligand 10. Oncol Lett (2020) 19(3):2495–501. doi: 10.3892/ol.2020.11326 PMC7039108 32194750

[168] Wang IC Chen YJ Hughes D Petrovic V Major ML Park HJ. Forkhead box M1 regulates the transcriptional network of genes essential for mitotic progression and genes encoding the SCF (Skp2-Cks1) ubiquitin ligase. Mol Cell Biol (2005) 25(24):10875–94. doi: 10.1128/MCB.25.24.10875-10894.2005 PMC1316960 16314512

[169] Zhang N Wei P Gong A Chiu WT Lee HT Colman H. FoxM1 promotes beta-catenin nuclear localization and controls Wnt target-gene expression and glioma tumorigenesis. Cancer Cell (2011) 20(4):427–42. doi: 10.1016/j.ccr.2011.08.016 PMC3199318 22014570

[170] Martin E Vivori C Rogalska M Herrero-Vicente J Valcarcel J. Alternative splicing regulation of cell-cycle genes by SPF45/SR140/CHERP complex controls cell proliferation. RNA (2021) 27(12):1557–76. doi: 10.1261/rna.078935.121 PMC8594467 34544891

[171] Fu Y Bai C Wang S Chen D Zhang P Wei H. AKT1 phosphorylates RBM17 to promote Sox2 transcription by modulating alternative splicing of FOXM1 to enhance cancer stem cell properties in colorectal cancer cells. FASEB J (2023) 37(1):e22707. doi: 10.1096/fj.202201255R 36520054

[172] Rather TB Parveiz I Bhat GA Rashid G Wani RA Khan IY. Evaluation of Forkhead BOX M1 (FOXM1) gene expression in colorectal cancer. Clin Exp Med (2022) 2022. doi: 10.1007/s10238-022-00929-7 36318377

[173] Fei BY He X Ma J Zhang M Chai R. FoxM1 is associated with metastasis in colorectal cancer through induction of the epithelial-mesenchymal transition. Oncol Lett (2017) 14(6):6553–61. doi: 10.3892/ol.2017.7022 PMC5686434 29163688

[174] Zhang HG Xu XW Shi XP Han BW Li ZH Ren WH. Overexpression of forkhead box protein M1 (FOXM1) plays a critical role in colorectal cancer. Clin Transl Oncol (2016) 18(5):527–32. doi: 10.1007/s12094-015-1400-1 26370421

[175] Lin F Worman HJ. Structural organization of the human gene encoding nuclear lamin A and nuclear lamin C. J Biol Chem (1993) 268(22):16321–6. 8344919

[176] Willis ND Cox TR Rahman-Casans SF Smits K Przyborski SA van den Brandt P. Lamin A/C is a risk biomarker in colorectal cancer. PloS One (2008) 3(8):e2988. doi: 10.1371/journal.pone.0002988 18714339 PMC2496895

[177] Pan YJ Huo FC Kang MJ Liu BW Wu MD Pei DS. Alternative splicing of HSPA12A pre-RNA by SRSF11 contributes to metastasis potential of colorectal cancer. Clin Transl Med (2022) 12(11):e1113. doi: 10.1002/ctm2.1113 36394206 PMC9670187

[178] Chao C Goluszko E Lee YT Kolokoltsov AA Davey RA Uchida T. Constitutively active CCK2 receptor splice variant increases Src-dependent HIF-1 alpha expression and tumor growth. Oncogene (2007) 26(7):1013–9. doi: 10.1038/sj.onc.1209862 16909104

[179] Lu Y Zhao X Li K Luo G Nie Y Shi Y. Thioredoxin-like protein 2 is overexpressed in colon cancer and promotes cancer cell metastasis by interaction with ran. Antioxid Redox Signal (2013) 19(9):899–911. doi: 10.1089/ars.2012.4736 23311631 PMC3763228

[180] Yu Z Zhang B Cui B Wang Y Han P Wang X. Identification of spliced variants of the proto-oncogene HDM2 in colorectal cancer. Cancer (2012) 118(4):1110–8. doi: 10.1002/cncr.26330 21761395

[181] Fanning AS Jameson BJ Jesaitis LA Anderson JM. The tight junction protein ZO-1 establishes a link between the transmembrane protein occludin and the actin cytoskeleton. J Biol Chem (1998) 273(45):29745–53. doi: 10.1074/jbc.273.45.29745 9792688

[182] Han F Yang B Zhou M Huang Q Mai M Huang Z. GLTSCR1 coordinates alternative splicing and transcription elongation of ZO1 to regulate colorectal cancer progression. J Mol Cell Biol (2022) 14(2):mjac009. doi: 10.1093/jmcb/mjac009 35218185 PMC9188103

[183] Wan L Yu W Shen E Sun W Liu Y Kong J. SRSF6-regulated alternative splicing that promotes tumour progression offers a therapy target for colorectal cancer. Gut (2019) 68(1):118–29. doi: 10.1136/gutjnl-2017-314983 29114070

[184] Heiner M Hui J Schreiner S Hung LH Bindereif A. HnRNP L-mediated regulation of mamMalian alternative splicing by interference with splice site recognition. RNA Biol (2010) 7(1):56–64. doi: 10.4161/rna.7.1.10402 19946215

[185] Kim YE Won M Lee SG Park C Song CH Kim KK. RBM47-regulated alternative splicing of TJP1 promotes actin stress fiber assembly during epithelial-to-mesenchymal transition. Oncogene (2019) 38(38):6521–36. doi: 10.1038/s41388-019-0892-5 31358901

[186] Jantscheff P Terracciano L Lowy A Glatz-Krieger K Grunert F Micheel B. Expression of CEACAM6 in resectable colorectal cancer: a factor of independent prognostic significance. J Clin Oncol (2003) 21(19):3638–46. doi: 10.1200/JCO.2003.55.135 14512395

[187] Kang WY Chen WT Wu MT Chai CY. The expression of CD66a and possible roles in colorectal adenoma and adenocarcinoma. Int J Colorectal Dis (2007) 22(8):869–74. doi: 10.1007/s00384-006-0247-x 17143599

[188] Barnett TR Drake L Pickle W 2nd. Human biliary glycoprotein gene: characterization of a family of novel alternatively spliced RNAs and their expressed proteins. Mol Cell Biol (1993) 13(2):1273–82. doi: 10.1128/mcb.13.2.1273-1282.1993 PMC359012 8423792

[189] Ieda J Yokoyama S Tamura K Takifuji K Hotta T Matsuda K. Re-expression of CEACAM1 long cytoplasmic domain isoform is associated with invasion and migration of colorectal cancer. Int J Cancer (2011) 129(6):1351–61. doi: 10.1002/ijc.26072 21413011

[190] Ling Y Kuang Y Chen LL Lao WF Zhu YR Wang LQ. A novel RON splice variant lacking exon 2 activates the PI3K/AKT pathway *via* PTEN phosphorylation in colorectal carcinoma cells. Oncotarget (2017) 8(24):39101–16. doi: 10.18632/oncotarget.16603 PMC5503598 28388571

[191] Wang D Lao WF Kuang YY Geng SM Mo LJ He C. A novel variant of the RON receptor tyrosine kinase derived from colorectal carcinoma cells which lacks tyrosine phosphorylation but induces cell migration. Exp Cell Res (2012) 318(20):2548–58. doi: 10.1016/j.yexcr.2012.08.006 22975341

[192] Rigillo G Belluti S Campani V Ragazzini G Ronzio M Miserocchi G. The NF-Y splicing signature controls hybrid EMT and ECM-related pathways to promote aggressiveness of colon cancer. Cancer Lett (2023) 567:216262. doi: 10.1016/j.canlet.2023.216262 37307894

[193] Krueger A Baumann S Krammer PH Kirchhoff S. FLICE-inhibitory proteins: regulators of death receptor-mediated apoptosis. Mol Cell Biol (2001) 21(24):8247–54. doi: 10.1128/MCB.21.24.8247-8254.2001 PMC99990 11713262

[194] Djerbi M Darreh-Shori T Zhivotovsky B Grandien A. Characterization of the human FLICE-inhibitory protein locus and comparison of the anti-apoptotic activity of four different flip isoforms. Scand J Immunol (2001) 54(1-2):180–9. doi: 10.1046/j.1365-3083.2001.00941.x 11439165

[195] Wilson TR McLaughlin KM McEwan M Sakai H Rogers KM Redmond KM. c-FLIP: a key regulator of colorectal cancer cell death. Cancer Res (2007) 67(12):5754–62. doi: 10.1158/0008-5472.CAN-06-3585 17575142

[196] Ryu BK Lee MG Chi SG Kim YW Park JH. Increased expression of cFLIP(L) in colonic adenocarcinoma. J Pathol (2001) 194(1):15–9. doi: 10.1002/path.835 11329136

[197] Law DJ Labut EM Adams RD Merchant JL. An isoform of ZBP-89 predisposes the colon to colitis. Nucleic Acids Res (2006) 34(5):1342–50. doi: 10.1093/nar/gkl022 PMC1390687 16517939

[198] Liu Y Huang W Gao X Kuang F. Regulation between two alternative splicing isoforms ZNF148(FL) and ZNF148(DeltaN), and their roles in the apoptosis and invasion of colorectal cancer. Pathol Res Pract (2019) 215(2):272–7. doi: 10.1016/j.prp.2018.10.036 30463804

[199] Denis V Cassagnard N Del Rio M Cornillot E Bec N Larroque C. Targeting the splicing isoforms of spleen tyrosine kinase affects the viability of colorectal cancer cells. PLoS One (2022) 17(9):e0274390. doi: 10.1371/journal.pone.0274390 36103569 PMC9473616

[200] Ni B Hu J Chen D Li L Chen D Wang J. Alternative splicing of spleen tyrosine kinase differentially regulates colorectal cancer progression. Oncol Lett (2016) 12(3):1737–44. doi: 10.3892/ol.2016.4858 PMC4998349 27602108

[201] Xu W Jing L Wang Q Lin CC Chen X Diao J. Bax-PGAM5L-Drp1 complex is required for intrinsic apoptosis execution. Oncotarget (2015) 6(30):30017–34. doi: 10.18632/oncotarget.5013 PMC4745779 26356820

[202] Wang Z Jiang H Chen S Du F Wang X. The mitochondrial phosphatase PGAM5 functions at the convergence point of multiple necrotic death pathways. Cell (2012) 148(1-2):228–43. doi: 10.1016/j.cell.2011.11.030 22265414

[203] Zhao H Ming T Tang S Ren S Yang H Liu M. Wnt signaling in colorectal cancer: pathogenic role and therapeutic target. Mol Cancer (2022) 21(1):144. doi: 10.1186/s12943-022-01616-7 35836256 PMC9281132

[204] Bueno MLP Saad STO Roversi FM. WNT5A in tumor development and progression: A comprehensive review. BioMed Pharmacother (2022) 155:113599. doi: 10.1016/j.biopha.2022.113599 36089446

[205] Katula KS Joyner-Powell NB Hsu CC Kuk A. Differential regulation of the mouse and human Wnt5a alternative promoters A and B. DNA Cell Biol (2012) 31(11):1585–97. doi: 10.1089/dna.2012.1698 PMC3482380 23046419

[206] Bauer M Benard J Gaasterland T Willert K Cappellen D. WNT5A encodes two isoforms with distinct functions in cancers. PloS One (2013) 8(11):e80526. doi: 10.1371/journal.pone.0080526 24260410 PMC3832467

[207] Huang TC Lee PT Wu MH Huang CC Ko CY Lee YC. Distinct roles and differential expression levels of Wnt5a mRNA isoforms in colorectal cancer cells. PloS One (2017) 12(8):e0181034. doi: 10.1371/journal.pone.0181034 28859077 PMC5578641

[208] Mo JS Alam KJ Kang IH Park WC Seo GS Choi SC. MicroRNA 196B regulates FAS-mediated apoptosis in colorectal cancer cells. Oncotarget (2015) 6(5):2843–55. doi: 10.18632/oncotarget.3066 PMC4413621 25605245

[209] Pryczynicz A Guzinska-Ustymowicz K Kemona A. Fas/FasL expression in colorectal cancer. An immunohistochemical study. Folia Histochem Cytobiol (2010) 48(3):425–9. doi: 10.2478/v10042-010-0058-3 21071349

[210] Vilys L Peciuliene I Jakubauskiene E Zinkeviciute R Makino Y Kanopka A. U2AF - Hypoxia-induced fas alternative splicing regulator. Exp Cell Res (2021) 399(1):112444. doi: 10.1016/j.yexcr.2020.112444 33347855

[211] Liu L Luo C Luo Y Chen L Liu Y Wang Y. MRPL33 and its splicing regulator hnRNPK are required for mitochondria function and implicated in tumor progression. Oncogene (2018) 37(1):86–94. doi: 10.1038/onc.2017.314 28869607

[212] Sillars-Hardebol AH Carvalho B Belien JA de Wit M Delis-van Diemen PM Tijssen M. BCL2L1 has a functional role in colorectal cancer and its protein expression is associated with chromosome 20q gain. J Pathol (2012) 226(3):442–50. doi: 10.1002/path.2983 22009326

[213] Joly F Fabbro M Follana P Lequesne J Medioni J Lesoin A. A phase II study of Navitoclax (ABT-263) as single agent in women heavily pretreated for recurrent epithelial ovarian cancer: The MONAVI - GINECO study. Gynecol Oncol (2022) 165(1):30–9. doi: 10.1016/j.ygyno.2022.01.021 35123771

[214] Ghaemi Z Mowla SJ Soltani BM. Novel splice variants of LINC00963 suppress colorectal cancer cell proliferation *via* miR-10a/miR-143/miR-217/miR-512-mediated regulation of PI3K/AKT and Wnt/beta-catenin signaling pathways. Biochim Biophys Acta Gene Regul Mech (2023) 1866(2):194921. doi: 10.1016/j.bbagrm.2023.194921 36804476

[215] Canavese M Ngo DT Maddern GJ Hardingham JE Price TJ Hauben E. Biology and therapeutic implications of VEGF-A splice isoforms and single-nucleotide polymorphisms in colorectal cancer. Int J Cancer (2017) 140(10):2183–91. doi: 10.1002/ijc.30567 27943279

[216] Watson CJ Webb NJ Bottomley MJ Brenchley PE. Identification of polymorphisms within the vascular endothelial growth factor (VEGF) gene: correlation with variation in VEGF protein production. Cytokine (2000) 12(8):1232–5. doi: 10.1006/cyto.2000.0692 10930302

[217] Woolard J Bevan HS Harper SJ Bates DO. Molecular diversity of VEGF-A as a regulator of its biological activity. Microcirculation (2009) 16(7):572–92. doi: 10.1080/10739680902997333 PMC2929464 19521900

[218] Des Guetz G Uzzan B Nicolas P Cucherat M Morere JF Benamouzig R. Microvessel density and VEGF expression are prognostic factors in colorectal cancer. Meta-analysis literature. Br J Cancer (2006) 94(12):1823–32. doi: 10.1038/sj.bjc.6603176 PMC2361355 16773076

[219] Saltz LB Clarke S Diaz-Rubio E Scheithauer W Figer A Wong R. Bevacizumab in combination with oxaliplatin-based chemotherapy as first-line therapy in metastatic colorectal cancer: a randomized phase III study. J Clin Oncol (2008) 26(12):2013–9. doi: 10.1200/JCO.2007.14.9930 18421054

[220] Diaz R Pena C Silva J Lorenzo Y Garcia V Garcia JM. VEGF165b and PEDF expression in human colorectal tumors: VEGF165b downregulation as a marker of poor prognosis. Int J Cancer (2008) 123(5):1060–7. doi: 10.1002/ijc.23619 18546269

[221] Eberhardt W Doller A Akool el S Pfeilschifter J. Modulation of mRNA stability as a novel therapeutic approach. Pharmacol Ther (2007) 114(1):56–73. doi: 10.1016/j.pharmthera.2007.01.002 17320967

[222] Hamdollah Zadeh MA Amin EM Hoareau-Aveilla C Domingo E Symonds KE Ye X. Alternative splicing of TIA-1 in human colon cancer regulates VEGF isoform expression, angiogenesis, tumour growth and bevacizumab resistance. Mol Oncol (2015) 9(1):167–78. doi: 10.1016/j.molonc.2014.07.017 PMC4286123 25224594

[223] Suswam EA Li YY Mahtani H King PH. Novel DNA-binding properties of the RNA-binding protein TIAR. Nucleic Acids Res (2005) 33(14):4507–18. doi: 10.1093/nar/gki763 PMC1184220 16091628

[224] Alnuaimi AR Nair VA Malhab LJB Abu-Gharbieh E Ranade AV Pintus G. Emerging role of caldesmon in cancer: A potential biomarker for colorectal cancer and other cancers. World J Gastrointest Oncol (2022) 14(9):1637–53. doi: 10.4251/wjgo.v14.i9.1637 PMC9516648 36187394

[225] Zheng PP van der Weiden M Kros JM. Differential expression of Hela-type caldesmon in tumour neovascularization: a new marker of angiogenic endothelial cells. J Pathol (2005) 205(3):408–14. doi: 10.1002/path.1700 15682433

[226] Kim KH Yeo SG Kim WK Kim DY Yeo HY Hong JP. Up-regulated expression of l-caldesmon associated with Malignancy of colorectal cancer. BMC Cancer (2012) 12:601. doi: 10.1186/1471-2407-12-601 23241148 PMC3572427

[227] Albuquerque RJ Hayashi T Cho WG Kleinman ME Dridi S Takeda A. Alternatively spliced vascular endothelial growth factor receptor-2 is an essential endogenous inhibitor of lymphatic vessel growth. Nat Med (2009) 15(9):1023–30. doi: 10.1038/nm.2018 PMC2882165 19668192

[228] Uehara H Cho Y Simonis J Cahoon J Archer B Luo L. Dual suppression of hemangiogenesis and lymphangiogenesis by splice-shifting morpholinos targeting vascular endothelial growth factor receptor 2 (KDR). FASEB J (2013) 27(1):76–85. doi: 10.1096/fj.12-213835 22997228 PMC3528308

[229] Stagg BC Uehara H Lambert N Rai R Gupta I Radmall B. Morpholino-mediated isoform modulation of vascular endothelial growth factor receptor-2 (VEGFR2) reduces colon cancer Xenograft growth. Cancers (Basel) (2014) 6(4):2330–42. doi: 10.3390/cancers6042330 PMC4276969 25534570

[230] Turano M Cammarota F Duraturo F Izzo P De Rosa M. A potential role of IL-6/IL-6R in the development and management of colon cancer. Membranes (Basel) (2021) 11(5):312. doi: 10.3390/membranes11050312 33923292 PMC8145725

[231] Schumertl T Lokau J Rose-John S Garbers C. Function and proteolytic generation of the soluble interleukin-6 receptor in health and disease. Biochim Biophys Acta Mol Cell Res (2022) 1869(1):119143. doi: 10.1016/j.bbamcr.2021.119143 34626681

[232] Chung YC Chang YF. Serum interleukin-6 levels reflect the disease status of colorectal cancer. J Surg Oncol (2003) 83(4):222–6. doi: 10.1002/jso.10269 12884234

[233] Zhu LQ Zhang L Zhang J Chang GL Liu G Yu DD. Evodiamine inhibits high-fat diet-induced colitis-associated cancer in mice through regulating the gut microbiota. J Integr Med (2021) 19(1):56–65. doi: 10.1016/j.joim.2020.11.001 33277208

[234] Khanna D Lin CJF Furst DE Wagner B Zucchetto M Raghu G. Long-term safety and efficacy of tocilizumab in early systemic sclerosis-interstitial lung disease: open-label extension of a phase 3 randomized controlled trial. Am J Respir Crit Care Med (2022) 205(6):674–84. doi: 10.1164/rccm.202103-0714OC 34851799

[235] Zhang S Chen B Wang B Chen H Li Y Cao Q. Effect of induction therapy with olamkicept vs placebo on clinical response in patients with active ulcerative colitis: A randomized clinical trial. JAMA (2023) 329(9):725–34. doi: 10.1001/jama.2023.1084 PMC9993185 36881032

[236] You M Yuan S Shi J Hou Y. PPARdelta signaling regulates colorectal cancer. Curr Pharm Des (2015) 21(21):2956–9. doi: 10.2174/1381612821666150514104035 26004416

[237] Saha L. Role of peroxisome proliferator-activated receptors alpha and gamma in gastric ulcer: An overview of experimental evidences. World J Gastrointest Pharmacol Ther (2015) 6(4):120–6. doi: 10.4292/wjgpt.v6.i4.120 PMC4635152 26558146

[238] Larsen LK Amri EZ Mandrup S Pacot C Kristiansen K. Genomic organization of the mouse peroxisome proliferator-activated receptor beta/delta gene: alternative promoter usage and splicing yield transcripts exhibiting differential translational efficiency. Biochem J (2002) 366(Pt 3):767–75. doi: 10.1042/BJ20011821 PMC1222822 12059785

[239] Michelet X Dyck L Hogan A Loftus RM Duquette D Wei K. Metabolic reprogramming of natural killer cells in obesity limits antitumor responses. Nat Immunol (2018) 19(12):1330–40. doi: 10.1038/s41590-018-0251-7 30420624

[240] Schumann T Adhikary T Wortmann A Finkernagel F Lieber S Schnitzer E. Deregulation of PPARbeta/delta target genes in tumor-associated macrophages by fatty acid ligands in the ovarian cancer microenvironment. Oncotarget (2015) 6(15):13416–33. doi: 10.18632/oncotarget.3826 PMC4537024 25968567

[241] Wagner N Wagner KD. PPAR Beta/Delta and the hallmarks of cancer. Cells (2020) 9(5):1133. doi: 10.3390/cells9051133 32375405 PMC7291220

[242] Wang R Li J Zhou X Mao Y Wang W Gao S. Single-cell genomic and transcriptomic landscapes of primary and metastatic colorectal cancer tumors. Genome Med (2022) 14(1):93. doi: 10.1186/s13073-022-01093-z 35974387 PMC9380328

[243] Gomez-Fernandez P Urtasun A Paton AW Paton JC Borrego F Dersh D. Long interleukin-22 binding protein isoform-1 is an intracellular activator of the unfolded protein response. Front Immunol (2018) 9:2934. doi: 10.3389/fimmu.2018.02934 30619294 PMC6302113

[244] Lim C Hong M Savan R. Human IL-22 binding protein isoforms act as a rheostat for IL-22 signaling. Sci Signal (2016) 9(447):ra95. doi: 10.1126/scisignal.aad9887 27678220

[245] Huber S Gagliani N Zenewicz LA Huber FJ Bosurgi L Hu B. IL-22BP is regulated by the inflammasome and modulates tumorigenesis in the intestine. Nature (2012) 491(7423):259–63. doi: 10.1038/nature11535 PMC3493690 23075849

[246] Pelczar P Witkowski M Perez LG Kempski J Hammel AG Brockmann L. A pathogenic role for T cell-derived IL-22BP in inflammatory bowel disease. Science (2016) 354(6310):358–62. doi: 10.1126/science.aah5903 27846573

[247] Martin JC Beriou G Heslan M Bossard C Jarry A Abidi A. IL-22BP is produced by eosinophils in human gut and blocks IL-22 protective actions during colitis. Mucosal Immunol (2016) 9(2):539–49. doi: 10.1038/mi.2015.83 26329427

[248] Zhang R Men K Zhang X Huang R Tian Y Zhou B. Delivery of a modified mRNA encoding IL-22 binding protein (IL-22BP) for colon cancer gene therapy. J BioMed Nanotechnol (2018) 14(7):1239–51. doi: 10.1166/jbn.2018.2577 29944098

[249] Manavalan JS Rossi PC Vlad G Piazza F Yarilina A Cortesini R. High expression of ILT3 and ILT4 is a general feature of tolerogenic dendritic cells. Transpl Immunol (2003) 11(3-4):245–58. doi: 10.1016/S0966-3274(03)00058-3 12967778

[250] Suciu-Foca N Feirt N Zhang QY Vlad G Liu Z Lin H. Soluble Ig-like transcript 3 inhibits tumor allograft rejection in humanized SCID mice and T cell responses in cancer patients. J Immunol (2007) 178(11):7432–41. doi: 10.4049/jimmunol.178.11.7432 17513794

[251] Liu J Lu CX Zhang F Lv W Liu C. Expression of ILT3 predicts poor prognosis and is inversely associated with infiltration of CD45RO+ T cells in patients with colorectal cancer. Pathol Res Pract (2018) 214(10):1621–5. doi: 10.1016/j.prp.2018.07.026 30126665

[252] Bluemlein K Gruning NM Feichtinger RG Lehrach H Kofler B Ralser M. No evidence for a shift in pyruvate kinase PKM1 to PKM2 expression during tumorigenesis. Oncotarget (2011) 2(5):393–400. doi: 10.18632/oncotarget.278 21789790 PMC3248187

[253] Park B Kim JY Riffey OF Dowker-Key P Bruckbauer A McLoughlin J. Pyruvate kinase M1 regulates butyrate metabolism in cancerous colonocytes. Sci Rep (2022) 12(1):8771. doi: 10.1038/s41598-022-12827-9 35610475 PMC9130307

[254] Zhu W Zhou BL Rong LJ Ye L Xu HJ Zhou Y. Roles of PTBP1 in alternative splicing, glycolysis, and oncogensis. J Zhejiang Univ Sci B (2020) 21(2):122–36. doi: 10.1631/jzus.B1900422 PMC7076342 32115910

[255] Lan Z Yao X Sun K Li A Liu S Wang X. The Interaction Between lncRNA SNHG6 and hnRNPA1 Contributes to the Growth of Colorectal Cancer by Enhancing Aerobic Glycolysis Through the Regulation of Alternative Splicing of PKM. Front Oncol (2020) 10:363. doi: 10.3389/fonc.2020.00363 32296635 PMC7136466

[256] Huang JZ Chen M Chen D Gao XC Zhu S Huang H. A peptide encoded by a putative lncRNA HOXB-AS3 suppresses colon cancer growth. Mol Cell (2017) 68(1):171–84 e6. doi: 10.1016/j.molcel.2017.09.015 28985503

[257] Zhao J Li J Hassan W Xu D Wang X Huang Z. Sam68 promotes aerobic glycolysis in colorectal cancer by regulating PKM2 alternative splicing. Ann Transl Med (2020) 8(7):459. doi: 10.21037/atm.2020.03.108 32395503 PMC7210197

[258] Taniguchi K Sugito N Kumazaki M Shinohara H Yamada N Nakagawa Y. MicroRNA-124 inhibits cancer cell growth through PTB1/PKM1/PKM2 feedback cascade in colorectal cancer. Cancer Lett (2015) 363(1):17–27. doi: 10.1016/j.canlet.2015.03.026 25818238

[259] Zheng H Zhang M Ke X Deng X Li D Wang Q. LncRNA XIST/miR-137 axis strengthens chemo-resistance and glycolysis of colorectal cancer cells by hindering transformation from PKM2 to PKM1. Cancer biomark (2021) 30(4):395–406. doi: 10.3233/CBM-201740 33386794 PMC12499988

[260] Han J Zhao Z Zhang N Yang Y Ma L Feng L. Transcriptional dysregulation of TRIM29 promotes colorectal cancer carcinogenesis *via* pyruvate kinase-mediated glucose metabolism. Aging (Albany NY) (2021) 13(4):5034–54. doi: 10.18632/aging.202414 PMC7950264 33495406

[261] Bellemare J Rouleau M Harvey M Tetu B Guillemette C. Alternative-splicing forms of the major phase II conjugating UGT1A gene negatively regulate glucuronidation in human carcinoma cell lines. Pharmacogenomics J (2010) 10(5):431–41. doi: 10.1038/tpj.2009.64 19997083

[262] Girard H Levesque E Bellemare J Journault K Caillier B Guillemette C. Genetic diversity at the UGT1 locus is amplified by a novel 3’ alternative splicing mechanism leading to nine additional UGT1A proteins that act as regulators of glucuronidation activity. Pharmacogenet Genomics (2007) 17(12):1077–89. doi: 10.1097/FPC.0b013e3282f1f118 18004212

[263] Levesque E Girard H Journault K Lepine J Guillemette C. Regulation of the UGT1A1 bilirubin-conjugating pathway: role of a new splicing event at the UGT1A locus. Hepatology (2007) 45(1):128–38. doi: 10.1002/hep.21464 17187418

[264] Audet-Delage Y Rouleau M Rouleau M Roberge J Miard S Picard F. Cross-talk between alternatively spliced UGT1A isoforms and colon cancer cell metabolism. Mol Pharmacol (2017) 91(3):167–77. doi: 10.1124/mol.116.106161 28049773

[265] Soupene E Kuypers FA. MamMalian long-chain acyl-CoA synthetases. Exp Biol Med (Maywood) (2008) 233(5):507–21. doi: 10.3181/0710-MR-287 PMC3377585 18375835

[266] Sanchez-Martinez R Cruz-Gil S Garcia-Alvarez MS Reglero G Ramirez de Molina A. Complementary ACSL isoforms contribute to a non-Warburg advantageous energetic status characterizing invasive colon cancer cells. Sci Rep (2017) 7(1):11143. doi: 10.1038/s41598-017-11612-3 28894242 PMC5593891

[267] Sanchez-Martinez R Cruz-Gil S Gomez de Cedron M Alvarez-Fernandez M Vargas T Molina S. A link between lipid metabolism and epithelial-mesenchymal transition provides a target for colon cancer therapy. Oncotarget (2015) 6(36):38719–36. doi: 10.18632/oncotarget.5340 PMC4770732 26451612

[268] Samuels-Lev Y O’Connor DJ Bergamaschi D Trigiante G Hsieh JK Zhong S. ASPP proteins specifically stimulate the apoptotic function of p53. Mol Cell (2001) 8(4):781–94. doi: 10.1016/s1097-2765(01)00367-7 11684014

[269] Wang Z Liu Y Takahashi M Van Hook K Kampa-Schittenhelm KM Sheppard BC. N terminus of ASPP2 binds to Ras and enhances Ras/Raf/MEK/ERK activation to promote oncogene-induced senescence. Proc Natl Acad Sci U.S.A. (2013) 110(1):312–7. doi: 10.1073/pnas.1201514110 PMC3538245 23248303

[270] Schittenhelm MM Walter B Tsintari V Federmann B Bajrami Saipi M Akmut F. Alternative splicing of the tumor suppressor ASPP2 results in a stress-inducible, oncogenic isoform prevalent in acute leukemia. EBioMedicine (2019) 42:340–51. doi: 10.1016/j.ebiom.2019.03.028 PMC6491939 30952616

[271] Rieger I Tsintari V Overkamp M Fend F Lopez CD Schittenhelm MM. ASPP2kappa is expressed in human colorectal carcinoma and promotes chemotherapy resistance and tumorigenesis. Front Mol Biosci (2021) 8:727203. doi: 10.3389/fmolb.2021.727203 34805267 PMC8602356

[272] Briones-Orta MA Avendano-Vazquez SE Aparicio-Bautista DI Coombes JD Weber GF Syn WK. Osteopontin splice variants and polymorphisms in cancer progression and prognosis. Biochim Biophys Acta Rev Cancer (2017) 1868(1):93–108 A. doi: 10.1016/j.bbcan.2017.02.005 28254527

[273] Chang S Huang J Niu H Wang J Si Y Bai Z. Epigenetic regulation of osteopontin splicing isoform c defines its role as a microenvironmental factor to promote the survival of colon cancer cells from 5-FU treatment. Cancer Cell Int (2020) 20:452. doi: 10.1186/s12935-020-01541-z 32944000 PMC7491101

[274] de Lau W Barker N Low TY Koo BK Li VS Teunissen H. Lgr5 homologues associate with Wnt receptors and mediate R-spondin signalling. Nature (2011) 476(7360):293–7. doi: 10.1038/nature10337 21727895

[275] Takahashi H Ishii H Nishida N Takemasa I Mizushima T Ikeda M. Significance of Lgr5(+ve) cancer stem cells in the colon and rectum. Ann Surg Oncol (2011) 18(4):1166–74. doi: 10.1245/s10434-010-1373-9 21125339

[276] Barker N Ridgway RA van Es JH van de Wetering M Begthel H van den Born M. Crypt stem cells as the cells-of-origin of intestinal cancer. Nature (2009) 457(7229):608–11. doi: 10.1038/nature07602 19092804

[277] Osawa H Takahashi H Nishimura J Ohta K Haraguchi N Hata T. Full-length LGR5-positive cells have chemoresistant characteristics in colorectal cancer. Br J Cancer (2016) 114(11):1251–60. doi: 10.1038/bjc.2016.112 PMC4891500 27140312

[278] Rot S Taubert H Bache M Greither T Wurl P Eckert AW. A novel splice variant of the stem cell marker LGR5/GPR49 is correlated with the risk of tumor-related death in soft-tissue sarcoma patients. BMC Cancer (2011) 11:429. doi: 10.1186/1471-2407-11-429 21978106 PMC3203099

[279] Xie T Geng J Wang Y Wang L Huang M Chen J. FOXM1 evokes 5-fluorouracil resistance in colorectal cancer depending on ABCC10. Oncotarget (2017) 8(5):8574–89. doi: 10.18632/oncotarget.14351 PMC5352423 28051999

[280] Varghese V Magnani L Harada-Shoji N Mauri F Szydlo RM Yao S. FOXM1 modulates 5-FU resistance in colorectal cancer through regulating TYMS expression. Sci Rep (2019) 9(1):1505. doi: 10.1038/s41598-018-38017-0 30728402 PMC6365533

[281] Nixon BR Sebag SC Glennon MS Hall EJ Kounlavong ES Freeman ML. Nuclear localized Raf1 isoform alters DNA-dependent protein kinase activity and the DNA damage response. FASEB J (2019) 33(1):1138–50. doi: 10.1096/fj.201800336R PMC6355065 30106602

[282] Gao Q Li XX Xu YM Zhang JZ Rong SD Qin YQ. IRE1alpha-targeting downregulates ABC transporters and overcomes drug resistance of colon cancer cells. Cancer Lett (2020) 476:67–74. doi: 10.1016/j.canlet.2020.02.007 32061752

[283] Hetz C Chevet E Harding HP. Targeting the unfolded protein response in disease. Nat Rev Drug Discovery (2013) 12(9):703–19. doi: 10.1038/nrd3976 23989796

[284] Calfon M Zeng H Urano F Till JH Hubbard SR Harding HP. IRE1 couples endoplasmic reticulum load to secretory capacity by processing the XBP-1 mRNA. Nature (2002) 415(6867):92–6. doi: 10.1038/415092a 11780124

[285] Xie Y Liu C Qin Y Chen J Fang J. Knockdown of IRE1a suppresses metastatic potential of colon cancer cells through inhibiting FN1-Src/FAK-GTPases signaling. Int J Biochem Cell Biol (2019) 114:105572. doi: 10.1016/j.biocel.2019.105572 31326465

[286] Guo Y Chen Y Ito H Watanabe A Ge X Kodama T. Identification and characterization of lin-28 homolog B (LIN28B) in human hepatocellular carcinoma. Gene (2006) 384:51–61. doi: 10.1016/j.gene.2006.07.011 16971064

[287] Mizuno R Chatterji P Andres S Hamilton K Simon L Foley SW. Differential regulation of LET-7 by LIN28B isoform-specific functions. Mol Cancer Res (2018) 16(3):403–16. doi: 10.1158/1541-7786.MCR-17-0514 PMC5835183 29330293

[288] Zhang X Ma D Xuan B Shi D He J Yu M. LncRNA CACClnc promotes chemoresistance of colorectal cancer by modulating alternative splicing of RAD51. Oncogene (2023) 42(17):1374–91. doi: 10.1038/s41388-023-02657-y 36906654

[289] Kim CJ Terado T Tambe Y Mukaisho KI Sugihara H Kawauchi A. Anti-oncogenic activities of cyclin D1b siRNA on human bladder cancer cells *via* induction of apoptosis and suppression of cancer cell stemness and invasiveness. Int J Oncol (2018) 52(1):231–40. doi: 10.3892/ijo.2017.4194 29115414

[290] Li Q Zeng C Liu H Yung KWY Chen C Xie Q. Protein-protein interaction inhibitor of SRPKs alters the splicing isoforms of VEGF and inhibits angiogenesis. iScience (2021) 24(5):102423. doi: 10.1016/j.isci.2021.102423 33997701 PMC8102418

[291] Shitara K Doi T Nagano O Imamura CK Ozeki T Ishii Y. Dose-escalation study for the targeting of CD44v(+) cancer stem cells by sulfasalazine in patients with advanced gastric cancer (EPOC1205). Gastric Cancer (2017) 20(2):341–9. doi: 10.1007/s10120-016-0610-8 27055559

[292] Tam BY Chiu K Chung H Bossard C Nguyen JD Creger E. The CLK inhibitor SM08502 induces anti-tumor activity and reduces Wnt pathway gene expression in gastrointestinal cancer models. Cancer Lett (2020) 473:186–97. doi: 10.1016/j.canlet.2019.09.009 31560935

